# The Differential Growth Inhibition of *Phytophthora* spp. Caused by the Rare Sugar Tagatose Is Associated With Species-Specific Metabolic and Transcriptional Changes

**DOI:** 10.3389/fmicb.2021.711545

**Published:** 2021-07-07

**Authors:** Abdessalem Chahed, Valentina Lazazzara, Marco Moretto, Andrea Nesler, Paola Elisa Corneo, Essaid Ait Barka, Ilaria Pertot, Gerardo Puopolo, Michele Perazzolli

**Affiliations:** ^1^Research and Innovation Centre, Fondazione Edmund Mach, San Michele all’Adige, Italy; ^2^Bi-PA nv, Londerzeel, Belgium; ^3^Department of Induced Resistance and Plant Bioprotection, University of Reims, Reims, France; ^4^Center Agriculture Food Environment (C3A), University of Trento, San Michele all’Adige, Italy

**Keywords:** rare sugar, *Phytophthora* spp., transcriptomics, gene expression level, targeted metabolomics, transcriptional reprogramming

## Abstract

Tagatose is a rare sugar with no negative impacts on human health and selective inhibitory effects on plant-associated microorganisms. Tagatose inhibited mycelial growth and negatively affected mitochondrial processes in *Phytophthora infestans*, but not in *Phytophthora cinnamomi*. The aim of this study was to elucidate metabolic changes and transcriptional reprogramming activated by *P. infestans* and *P. cinnamomi* in response to tagatose, in order to clarify the differential inhibitory mechanisms of tagatose and the species-specific reactions to this rare sugar. *Phytophthora infestans* and *P. cinnamomi* activated distinct metabolic and transcriptional changes in response to the rare sugar. Tagatose negatively affected mycelial growth, sugar content and amino acid content in *P. infestans* with a severe transcriptional reprogramming that included the downregulation of genes involved in transport, sugar metabolism, signal transduction, and growth-related process. Conversely, tagatose incubation upregulated genes related to transport, energy metabolism, sugar metabolism and oxidative stress in *P. cinnamomi* with no negative effects on mycelial growth, sugar content and amino acid content. Differential inhibitory effects of tagatose on *Phytophthora* spp. were associated with an attempted reaction of *P. infestans*, which was not sufficient to attenuate the negative impacts of the rare sugar and with an efficient response of *P. cinnamomi* with the reprogramming of multiple metabolic processes, such as genes related to glucose transport, pentose metabolism, tricarboxylic acid cycle, reactive oxygen species detoxification, mitochondrial and alternative respiration processes. Knowledge on the differential response of *Phytophthora* spp. to tagatose represent a step forward in the understanding functional roles of rare sugars.

## Introduction

Rare sugars have been defined as monosaccharides and their derivatives that rarely exist in nature, such as tagatose, allose, sorbose, xylulose, and xylitol (Granström et al., 2004). The potential functional properties of rare sugars are underestimated due to their limited availability in nature (Li et al., 2013). However, the implementation of novel enzymatic and microbial processes lowered the cost of rare sugar synthesis and extended their use in various industrial and scientific fields, such as agriculture, human nutrition and medicine (Granström et al., 2004; Izumori, 2006; Li et al., 2013). Among rare sugars, tagatose is a ketohexose that was found at low concentrations in many foods, such as apple, pineapple, orange and milk (Vastenavond et al., 2011). Since tagatose does not have negative impacts on human health, it was ‘generally recognized as safe’ by the Food and Drug Administration and it is used as low-calorie sweetener in several countries (Levin, 2002; Vastenavond et al., 2011). Tagatose can be utilized as a carbohydrate source by only a restricted number of microorganisms, such as *Exiguobacterium* spp., *Lactobacillus* spp., and *Lactococcus* spp. (Raichand et al., 2012; Martinussen et al., 2013; Van der Heiden et al., 2013; Wu and Shah, 2017). On the other hand, tagatose is not catabolised by some human-associated microorganisms, such as *Bacillus cereus*, *Escherichia coli*, *Listeria monocytogenes*, *Staphylococcus aureus*, and *Yersinia enterocolitica* (Bautista et al., 2000), indicating selective nutritional or anti-nutritional properties for specific microbial taxa. For example, tagatose inhibited the growth of *Streptococcus mutans* (Hasibul et al., 2018) and *Salmonella enterica* serovar Typhimurium (Lobete et al., 2017) and decreased the gene expression (e.g., glucosyltransferase, fructosidase, and phosphotransferase encoding genes) and the enzymatic activity (e.g., glucosyltransferase) of sugar metabolism in *S. mutans* (Hasibul et al., 2018). Conversely, tagatose enhanced the growth of *Lactobacillus rhamnosus* and upregulated genes associated with sugar metabolism (e.g., phosphotransferase encoding genes, phosphofructokinase and tagatose-6-phosphate kinase) (Koh et al., 2013), suggesting specific impacts of rare sugars on the microbial metabolism.

Tagatose inhibited the growth of several causal agents of plant diseases, such as *Phytophthora infestans* (tomato and potato late blight) (Chahed et al., 2020), *Plasmopara viticola* (grapevine downy mildew) and *Erysiphe necator* (grapevine powdery mildew) (Perazzolli et al., 2020), *Hyaloperonospora parasitica* (cabbage downy mildew) and *Oidium violae* (tomato powdery mildew) (Mochizuki et al., 2020; Mijailovic et al., 2021). Tagatose showed also possible prebiotic effects on the phyllosphere microbiota and shifted the proportions of potential beneficial and potential pathogenic microorganisms by a selective nutritional or anti-nutritional effect on plant-associated microorganisms (Perazzolli et al., 2020). Differential effects were also found on *Trichoderma* spp. growth, where tagatose supported the growth of *T. harzianum* and *T. pleuroticola*, but not that of *T. pleurotum* (Komon-Zelazowska et al., 2007), confirming the selective effect of tagatose on plant-associated microorganisms. In particular, tagatose inhibited mycelial growth with severe mitochondrial alterations and stress-related gene modulation in *P. infestans*, but not in *P. cinnamomi* (Chahed et al., 2020), indicating species-specific responses to this rare sugar. Further studies are therefore required, in order to better understand the metabolic and transcriptional reprogramming responsible for the differential inhibitory effects of tagatose on plant-associated microorganisms.

Members of the *Phytophthora* genus are widespread causal agents of destructive diseases for different plant species (Kamoun, 2000). In particular, *P. infestans* and *P. cinnamomi* are two of the most important phytopathogenic oomycetes (Kamoun et al., 2015) and are responsible for severe economic losses in agricultural, horticultural and forest production (Hardham, 2005; Fry et al., 2015; Kamoun et al., 2015). The control of phytopathogenic oomycetes requires the use of chemical fungicides (Judelson and Blanco, 2005) with potential negative effects on the environment and human health (Fantke et al., 2012). Tagatose could represent a valid alternative to chemical fungicides, thanks to its inhibitory properties against *P. infestans* (Chahed et al., 2020) and the absence of negative impacts on human health (Ohara et al., 2008), but further studies are needed to clarify its impacts on metabolic and transcriptional regulations of *Phytophthora* spp. For example, the fungicide ethylicin severely affected amino acid and sugar metabolic processes in *P. infestans* (Zhang et al., 2020). Likewise, changes in the sugar, amino acid and organic acid content were observed in *P. infestans* isolates resistant to the fungicide metalxyl, indicating that fungicide resistance pathways are linked to the modulation of fatty acid biosynthesis and glycerophospholipid metabolism (Maridueña-Zavala et al., 2017). Transcriptional reprogramming in *Phytophthora* spp. exposed to biofungicides was also found and the natural product melatonin inhibited *P. infestans* growth by the downregulation of genes related to amino acid and sugar metabolism (Zhang et al., 2017). Similarly, the growth inhibition of *P. infestans* caused by *Lysobacter capsici* AZ78 (Tomada et al., 2017) and that of *Phytophthora sojae* caused by *Bacillus amyloliquefaciens* JDF3 and *B. subtilis* RSS (Liu et al., 2019) was associated with the downregulation of genes related to protein and sugar metabolism, indicating that complex metabolic responses are activated in *Phytophthora* spp. in response to biological or chemical growth inhibitors. The aim of this study was to elucidate the metabolic changes and transcriptional reprogramming activated by *Phytophthora* spp. in response to tagatose incubation, in order to clarify the differential inhibitory mechanisms of tagatose and the molecular determinants of the species-specific reaction to this rare sugar.

## Materials and Methods

### *Phytophthora* spp. Growth Conditions and Tagatose Incubation

*Phytophthora infestans* strain VB3 and *P. cinnamomi* strain CBS 144.22 were stored in glycerol at −80°C in the fungal collection of the Fondazione Edmund Mach and they are available upon request. *P. infestans* and *P. cinnamomi* were grown in Petri dishes on pea agar medium (PAM; 12.5% frozen peas and 1.2% agar in distilled water) at 18 and 25°C in dark conditions, respectively (Chahed et al., 2020). The stock solution (50 g/L in distilled water) of tagatose (Bi-PA, Londerzeel, Belgium) was filter sterilized and added at the final concentration of 5 g/L on PAM shortly before *Phytophthora* spp. inoculation, as reported by Chahed et al. (2020).

*Phytophthora* spp. growth was assessed as previously described (Chahed et al., 2020). Briefly, a plug (5 mm diameter and 2 mm height) of a 14 days-old colony was placed at the center of each dish (90 mm diameter) on PAM in the absence (control) or presence of 5 g/L tagatose (tagatose-incubated). The radial growth of *P. infestans* and *P. cinnamomi* was assessed at 4 and at 10 days after incubation (DAI) at 18 and 25°C, respectively, calculated as the average of the two perpendicular diameters of the colony, minus the plug diameter and the result divided by two. Ten replicates (dishes) were used for each treatment and the experiment was carried out twice.

For metabolic and transcriptomic analyses, a plug (5 mm diameter and 2 mm height) of a 14 days-old colony was placed on PAM covered with sterile cellophane layers in the absence (control) and presence of 5 g/L tagatose. *P. infestans* and *P. cinnamomi* samples were collected at 4 and at 10 DAI after incubation at 18 and 25°C, respectively, as previously described (Chahed et al., 2020). Briefly, mycelium samples were collected with a sterile forceps, transferred in a sterile 50 mL-tube, immediately frozen in liquid nitrogen and stored at −80°C. Samples were crushed using a mixer mill disruptor (MM200, Retsch, Haan, Germany) at 25 Hz for 45 s with sterile steel jars and beads refrigerated in liquid nitrogen.

### Sugar and Amino Acid Quantification

Ground *Phytophthora* spp. mycelium (500 mg) or uninoculated PAM (500 mg; Supplementary Table 1) were dissolved in 25 mL of ultrapure water, filtered through a 0.22 μm PTFE membrane (Sartorius, Göttingen, Germany) and used for sugar and amino acid quantification (Chemistry Unit at Fondazione Edmund Mach).

The sugar content (arabinose, fructose, glucose, isomaltose, lactose, maltose, mannose, melibiose, rhamnose, ribose, tagatose, trehalose, and xylose) was assessed by ion chromatography (Cataldi et al., 2000) and it was expressed as quantity of each sugar per unit of mycelium fresh weight (mg/kg), using a calibration curve of each pure sugar (Sigma-Aldrich, Merc, Kenilworth, NJ, United States) dissolved in ultrapure water within a range between 0.4 and 40 μg/mL. Briefly, *Phytophthora* spp. mycelium samples, uninoculated PAM samples and calibration curves of each pure sugar were analysed with an ionic chromatograph ICS 5000 (Dionex-Thermo Scientific, Waltham, MA, United States), equipped with an autosampler, a quaternary gradient pump, a column oven and a pulsed amperometric detector with a gold working electrode and a palladium counter electrode. The separation was obtained by injecting 5 μL of each *Phytophthora* spp. mycelium sample, uninoculated PAM sample or pure sugar onto a CarboPac PA200 3 mm × 250 mm analytical column (Dionex, Thermo Scientific, Waltham, MA, United States), preceded by a CarboPac PA200 3 mm × 50 mm guard column (Dionex, Thermo Scientific), with a NaOH gradient (from 1 to 30 mM) at 0.4 mL/min flow rate.

The amino acid content (aminobutyric acid, ethanolamine, glycine, glutamic acid, aspartic acid, alanine, arginine, asparagine, citrulline, phenylalanine, glutamine, isoleucine, histidine, leucine, lysine, ornithine, serine, tyrosine, threonine, valine, tryptophan, and methionine) was assessed by high performance liquid chromatography (HPLC) (Hill et al., 1979) and it was expressed as quantity of each amino acid per unit of mycelium fresh weight (mg/kg), using a calibration curve of each pure amino acid (Sigma-Aldrich, Merck) dissolved in ultrapure water within a range between 0.1 and 50 μg/mL. Briefly, *Phytophthora* spp. mycelium samples, uninoculated PAM samples and calibration curves of each amino acid were analyzed with a HPLC instrument (1100 series, Agilent Technologies, Santa Clara, CA, United States) equipped with a degasser, a quaternary gradient pump, an autosampler, a thermostatted column oven and a Fluorimetric Detector (FLD). The chromatographic separation of amino acids was obtained by injecting 5 μL of each *Phytophthora* spp. mycelium sample, uninoculated PAM sample or pure amino acid onto a Chromolith Performance RP-18e column (100 mm × 4.6 mm) and a guard RP-18e column (10 mm × 4.6 mm; Merck, Kenilworth, NJ, United States) kept at 40°C with an eluent flow of 2.0 mL/min. The FLD was set at 336 nm as excitation and at 445 nm as emission wavelength.

For sugar and amino acid quantification, three replicates (each replicate obtained as a pool of ten dishes) were analyzed for each *Phytophthora* spp., treatment and time point, and the experiment was carried out twice.

### RNA Extraction, Sequencing and Mapping to Reference Genomes

Total RNA was extracted from ground *Phytophthora* spp. mycelium (100 mg) using the Spectrum Plant total RNA kit (Sigma-Aldrich, Merck) with an on-column DNase treatment using the RNase-Free DNase Set (Qiagen, Hilden, Germany). Total RNA was quantified using a Qubit (Thermo Fisher Scientific) and RNA quality was checked using a Bioanalyzer 2100 (Agilent Technologies). Four replicates (each replicate obtained as a pool of 10 dishes) were analyzed for each *Phytophthora* spp., treatment and time point. RNA samples were subjected to RNA-Seq library construction, using the TruSeq Stranded Total RNA Library Prep Plant protocol (Illumina, San Diego, CA, United States) with rRNA depletion with the Ribo-Zero Plant kit (Tomada et al., 2017) according to the manufacturer’s instructions. Paired-end reads of 150 bases were obtained using a HiSeq 2500 (Illumina) at the Institute of Applied Genomics (Udine, Italy).

Raw Illumina reads were cleaned and filtered using Trimmomatic program version 0.36 (Bolger et al., 2014) and low-quality bases with an average Phred quality score lower than 15 in a sliding window of four base were removed. Reads shorter than 36 bases in length were removed from the analysis and quality check of raw reads was performed using Fast QC version 0.11.7. Read pairs of *P. infestans* samples were aligned to the *P. infestans* ASM14294v1 genome^1^ using the STAR V2.7 program (Dobin et al., 2013), while those of *P. cinnamomi* samples were aligned to *P. cinnamomi* V1.0 genome^2^. Counts of unambiguously mapped read pairs were obtained during the alignment with the STAR V2.7 program. Putative orthologous genes of *P. infestans* and *P. cinnamomi* (orthologous genes) were identified by reciprocal best BLAST hit using the BLAST+ 2.7.1 program (Camacho et al., 2009) with a threshold of 70% identity and 50% alignment length on amino acid sequences, in order to better analyze the differential *Phytophthora* spp. response.

### Identification and Functional Annotation of Differentially Expressed Genes

Differentially expressed genes (DEGs) were identified with the Limma-Voom package (Law et al., 2014) which estimates the mean–variance relationship of log counts, generating a precision weight for each observation that is fed into the Limma empirical Bayes analysis pipeline (Smyth, 2004). Pearson correlation analysis and Volcano Plot analysis (Patterson et al., 2006) were carried out using the Python programming language and the matplotlib package (Hunter, 2007) and a double cutoff on *P*-value (*P* ≤ 0.01) and minimum Log_2_ fold change (FC) of one [Log_2_ (FC) ≥ 1 or Log_2_ (FC) ≤ −1] was used to select DEGs, as previously reported (Tomada et al., 2017; Shen et al., 2020). Four pairwise comparisons were analyzed, in order to identify DEGs in each *Phytophthora* spp.: tagatose-incubated *vs.* control of *P. infestans* at 4 DAI, tagatose-incubated *vs.* control of *P. infestans* at 10 DAI, tagatose-incubated vs. control of *P. cinnamomi* at 4 DAI, tagatose-incubated *vs.* control of *P. cinnamomi* at 10 DAI. The distribution of *P. infestans* and *P. cinnamomi* DEGs was summarized using Venn diagram^3^ and DEGs were grouped in upregulated genes [*P* ≤ 0.01 and Log_2_ (FC) ≥ 1] or downregulated genes [*P* ≤ 0.01 and Log_2_ (FC) ≤ −1] at both time points (4 and 10 DAI cluster) and exclusively at 4 DAI (4 DAI cluster) or at 10 DAI (10 DAI cluster) for each *Phytophthora* spp. The heat map diagram of Log_2_-transformed FC values of *Phytophthora* spp. DEGs was visualized using the Java Treeview tool (Saldanha, 2004).

On the list of *Phytophthora* orthologous genes (8,908 in total), orthologous DEGs were identified imposing a *P*-value lower than 0.01 and minimum Log_2_ FC of one using a Volcano Plot (Patterson et al., 2006) with the Python programming language and the matplotlib package (Hunter, 2007) in the pairwise comparisons between tagatose-incubated samples and control samples at 4 DAI and at 10 DAI for each *Phytophthora* spp. Contrasts were defined in order to identify genes that respond differently in *P. infestans* compared to *P. cinnamomi* in the comparison between control and tagatose-incubated samples at 4 and at 10 DAI. Orthologous DEGs were then grouped in 16 clusters (defined by a four-letter code) based on their upregulation (U) or downregulation (D) in tagatose-incubated samples compared to control samples in the two *Phytophthora* spp. at 4 and at 10 DAI (e.g., UUDD cluster includes genes upregulated in *P. infestans* at 4 and 10 DAI and downregulated in *P. cinnamomi* at 4 and 10 DAI).

A principal component analysis (PCA) was performed using the Python programming language and the scikit.learn Python package^4^ on Limma-normalized expression values. Gene sequences of *P. infestans* and *P. cinnamomi* were aligned against the UniProtKB database (downloaded on May 2019^5^) using a BLAST-X search and the three or five best protein hits with an *E*-value lower than 1 × 10^–5^ were selected for functional annotation. In particular, DEGs were annotated based on the *Phytophthora* spp. protein description resulted by BLAST-X search and grouped into 17 functional categories according to the previous literature. *Phytophthora* spp. genes were further annotated using ARGOT2 function prediction tool for the Gene Ontology functional annotation (Falda et al., 2012). Gene ontology (GO) terms significantly overrepresented (*P* ≤ 0.05, Benjamin and Hochberg FDR correction) in the DEG lists in comparison to the whole transcriptome of the respective *Phytophthora* spp. were identified using the Biological Networks Gene Ontology (BiNGO) tool (Maere et al., 2005) and biological networks were visualized with Cytoscape version 3.7.2 (Shannon et al., 2003).

### Gene Expression Analysis by Quantitative Real-Time RT-PCR

*Phytophthora* spp. genes were selected for quantitative real-time PCR (qPCR) analysis (Supplementary Table 2). A primer pair compatible for the *P. infestans* and *P. cinnamomi* sequence was designed on conserved coding regions in the case of orthologous genes. The first strand cDNA was synthesized from 1 μg of DNase-treated RNA using Superscript III (Invitrogen, Thermo Fisher Scientific) and the oligo-dT primer. qPCR reactions were carried out with Platinum SYBR Green qPCR SuperMix-UDG (Invitrogen, Thermo Fisher Scientific) and specific primers using the Light Cycler 480 (Roche Diagnostics, Mannheim, Germany), as previously described (Chahed et al., 2020). Briefly, the PCR conditions were: 50°C for 2 min and 95°C for 2 min as initial steps, followed by 50 cycles of 95°C for 15 s and 60°C for 1 min. Each sample was examined in three technical replicates and dissociation curves were analyzed to verify the specificity of each amplification reaction. Light Cycler 480 SV1.5.0 software (Roche) was used to extract Ct values based on the second derivative calculation and LinReg software was used to calculate reaction efficiencies (Ruijter et al., 2009). The relative expression level (FC) of each gene was calculated according to the Pfaffl equation (Pfaffl, 2001) for tagatose-incubated samples compared to the respective control samples (calibrator) for each *Phytophthora* spp. and time point. The gene encoding β-tubulin (*tub-b*) was used as constitutive gene for the expression level normalization (Yan and Liou, 2006), because its expression was not significantly affected by the treatments (Supplementary Table 2). Four replicates (each replicate obtained as a pool of ten dishes) were analyzed for each *Phytophthora* spp., treatment and time point.

### Statistical Analysis

Mycelial growth, sugar and amino acid data were analyzed with Statistica 13.1 software (TIBCO Software, Palo Alto, CA, United States). Normal distribution (Kolmogorov–Smirnov test, *P* > 0.05) and variance homogeneity of the data (Levene’s tests, *P* > 0.05) were checked and parametric tests were used when both assumptions were respected. Each experimental repetition was analyzed singularly and a two-way analysis of variance (two-way ANOVA) was used to demonstrate non-significant differences between the two experiments (*P* > 0.05). Data from the two experimental repetitions were pooled and significant differences between tagatose-incubated samples and control samples were assessed with the Student’s *t*-test (*P* ≤ 0.05) for each *Phytophthora* spp. and time point.

## Results

### Tagatose Affects the Mycelial Growth, Sugar Content and Amino Acid Content in *Phytophthora infestans*, but Not in *P. cinnamomi*

**FIGURE 1:**
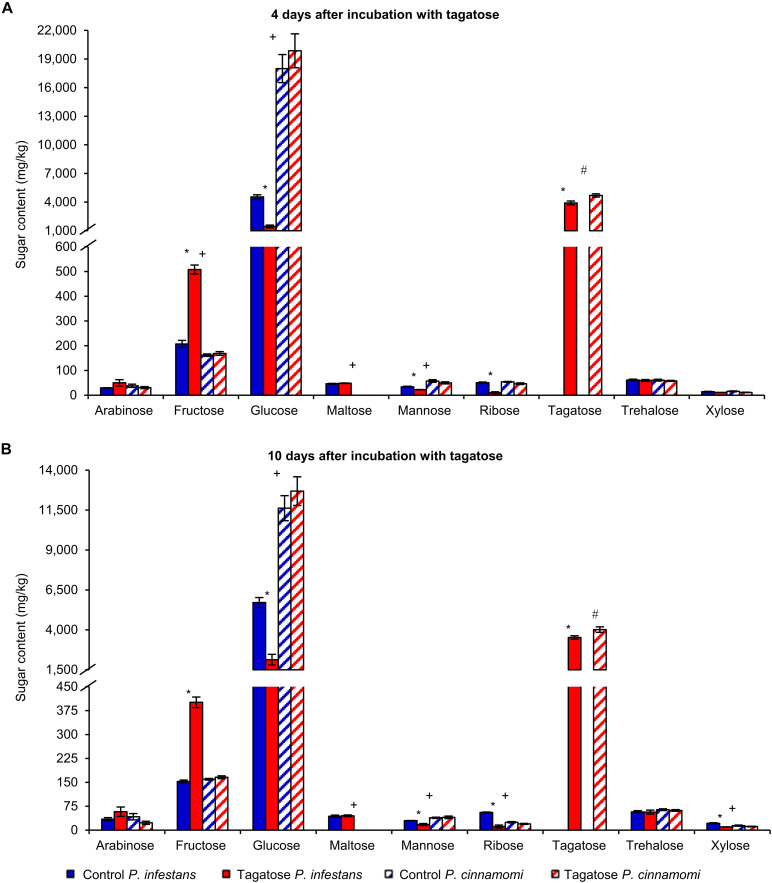
Effect of tagatose on *Phytophthora* spp. sugar content. *Phytophthora infestans* (solid bars) and *P. cinnamomi* (stripped bars) sugar content was quantified at 4 **(A)** and 10 **(B)** days after incubation (DAI) on pea agar medium in the absence (control, blue) and presence of tagatose (red). The two-way analysis of variance (two-way ANOVA) showed no significant differences between the two experimental repetitions (*P* > 0.05, three replicates per experiment) and data from the two experiments were pooled. Mean and standard error values of six replicates from the two experiments are presented for each treatment. For each sugar and time point, significant differences between tagatose-incubated samples and control samples are marked with an asterisk (*) for *P. infestans* and a hashtag (#) for *P. cinnamomi*, according to the Student’s *t*-test (*P* ≤ 0.05). Significant differences between *P. infestans* control and *P. cinnamomi* control for each sugar are marked with a plus sign (+), according to the Student’s *t*-test (*P* ≤ 0.05). Isomaltose, lactose, melibiose and rhamnose were not detected and omitted from the chart.

Tagatose inhibited the growth of *P. infestans* and not that of *P. cinnamomi* (Table 1 and Supplementary Figure 1) at 4 and at 10 DAI on PAM. In order to investigate impacts of tagatose on *Phytophthora* spp. metabolism, sugar content and amino acid content of *P. infestans* and *P. cinnamomi* mycelium were analyzed at 4 and at 10 DAI on PAM in the absence (control) and presence of tagatose (tagatose-incubated) by ion chromatography and HPLC, respectively (Figure 1 and Supplementary Figure 2). In *P. infestans*, tagatose incubation decreased the glucose, mannose and ribose content at 4 and at 10 DAI and the xylose content at 10 DAI compared to the respective control samples (Figures 1A,B). Conversely, the fructose content of *P. infestans* was higher in tagatose-incubated samples compared to control samples at 4 and at 10 DAI. In *P. cinnamomi*, the content of each sugar was comparable in tagatose-incubated samples and control samples at 4 and at 10 DAI (Figures 1A,B). As expected, tagatose was found only in tagatose-incubated samples of *P. infestans* and *P. cinnamomi* at both time points, but not in the respective control samples. The sugar content in control samples differed between *P. infestans* and *P. cinnamomi* in terms of glucose, maltose and mannose content at 4 and at 10 DAI, fructose content at 4 DAI, ribose and xylose content at 10 DAI.

**TABLE 1:** Effect of tagatose on *Phytophthora* spp. growth.

Species	Mycelial growth (mm)					
	4 DAI			10 DAI		
	Control	Tagatose	Significance	Control	Tagatose	Significance
*Phytophthora infestans*	8.80 ± 0.44	1.39 ± 0.39	*	19.14 ± 0.69	5.02 ± 1.34	*
*Phytophthora cinnamomi*	32.40 ± 1.28	30.98 ± 1.91		38.5 ± 0.00	38.5 ± 0.00	

*Phytophthora infestans and P. cinnamomi growth (mm) was assessed 4 and 10 days after incubation (DAI) on pea agar medium in the absence (control) and presence of tagatose. The two-way analysis of variance (two-way ANOVA) showed no significant differences between the two experimental repetitions (P > 0.05, 10 replicates per experiment) and data from the two experiments were pooled. Mean and standard error values of 20 replicates from the two experiments are reported for each treatment. Significant differences between tagatose-incubated samples and control samples are marked with an asterisk for each Phytophthora spp. and time point, according to the Student’s t-test (P ≤ 0.05).*

The amino acid content was affected by tagatose in *P. infestans* at 4 and at 10 DAI, but only slightly in *P. cinnamomi* at 4 DAI (Supplementary Figures 2A,B). In *P. infestans*, tagatose incubation decreased the content of 14 amino acids at 4 and at 10 DAI (aminobutyric acid, ethanolamine, glycine, aspartic acid, arginine, asparagine, phenylalanine, glutamine, isoleucine, leucine, ornithine, serine, tryptophan, and methionine), the content of two amino acids at 4 DAI (alanine and histidine) and the content of two amino acids at 10 DAI (lysine and tyrosine) compared to the respective control samples. Conversely, the glutamic acid content at 4 and at 10 DAI, or the histidine content at 10 DAI, was higher in tagatose-incubated samples compared to control *P. infestans* samples. In *P. cinnamomi*, the amino acid content was comparable in tagatose-incubated samples and control samples at both time points, except for the lower glutamic acid and glutamine content found in tagatose-incubated samples compared to control samples at 4 DAI.

### RNA-Seq Analysis Reveals Species-Specific Response of *Phytophthora* spp. Genes to Tagatose Incubation

To clarify *Phytophthora* spp. transcriptional regulations in response to tagatose incubation, RNA-Seq analysis was carried out on control and tagatose-incubated samples of *P. infestans* and *P. cinnamomi* at 4 and at 10 DAI. Sequences of the 32 samples [two *Phytophthora* spp. (*P. infestans* and *P. cinnamomi*), two incubation conditions (tagatose-incubated and control), two time points (4 and 10 DAI) and four replicates] were obtained (BioProject number PRJNA622764) and the total number of read pairs that mapped uniquely to the *P. infestans* and *P. cinnamomi* genome ranged from 7,862,604 to 12,711,308 and from 4,197,875 to 9,047,846 for each replicate, respectively (Supplementary Table 3). The expression level of *P. infestans* and *P. cinnamomi* genes was assessed (Supplementary Tables 4, 5) and high Pearson correlation values among replicates were found (Supplementary Figures 3–10 and Supplementary Tables 6–8). Global effects of tagatose incubation on the *Phytophthora* spp. transcriptome were visualized by PCA, which discriminated *P. infestans* samples according to the incubation condition in the first principal component (first PC, 81.6%) and according to the time point in the second PC (5.22%; Supplementary Figure 11A). In the case of *P. cinnamomi*, PCA classified samples according to the time point in the first PC (54.15%) and according to the incubation condition in the second PC (7.73%; Supplementary Figure 11B). Moreover, the PCA on the putative orthologous genes of *P. infestans* and *P. cinnamomi* (8,908 orthologous genes, in total) discriminated samples according to the species in the first PC (72.48%) and highlighted changes between tagatose-incubated samples and control samples of *P. infestans* on the second PC (Supplementary Figure 11C).

**FIGURE 2:**
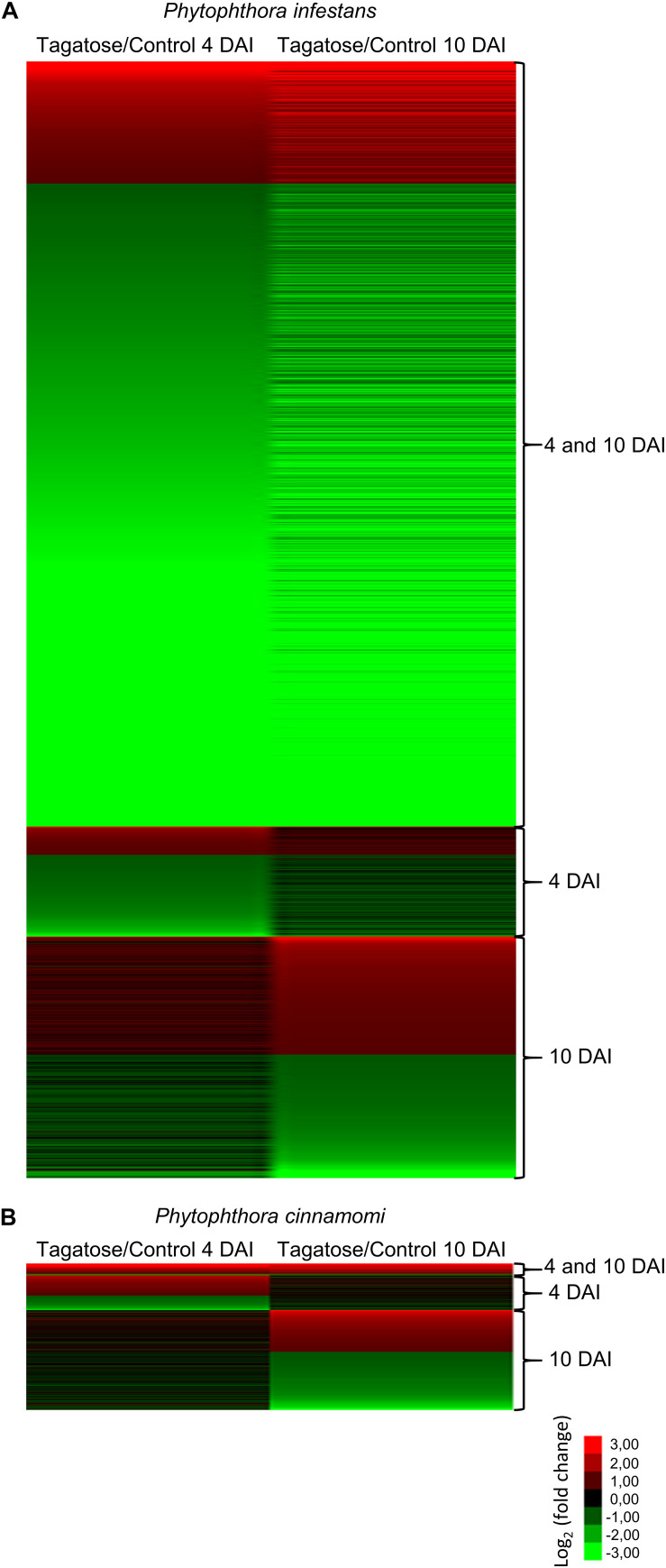
Clustering of genes modulated by tagatose in *Phytophthora* spp. Heat map diagram of fold change values of differentially expressed gens (DEGs) identified in *Phytophthora infestans* **(A)** and *P. cinnamomi* **(B)** at 4 and 10 days after incubation (DAI) on pea agar medium in the presence of tagatose compared to the respective control samples grown in the absence of tagatose. For each *Phytophthora* spp., DEGs were grouped in genes significantly upregulated or downregulated at both time points (4 and 10 DAI cluster) and exclusively modulated at 4 DAI (4 DAI cluster) or at 10 DAI (10 DAI cluster). The heat map diagram was visualized using Java Treeview according to the color scale legend shown.

Tagatose incubation resulted in the modulation of 3,901 in *P. infestans* (Supplementary Table 9) and 512 DEGs in *P. cinnamomi* (Supplementary Table 10), according to the Volcano Plot analysis (*P* ≤ 0.01 and minimum Log_2_ (FC) of one; Supplementary Figure 12). A large fraction (75.9%) of *P. infestans* DEGs and almost half (50.5%) of *P. cinnamomi* DEGs were downregulated by tagatose (Figure 2). DEGs were grouped in upregulated or downregulated genes at both time points (4 and 10 DAI cluster) and exclusively at 4 DAI (4 DAI cluster) or at 10 DAI (10 DAI cluster) for each *Phytophthora* spp. (Figure 2 and Supplementary Figure 13). Moreover, 2,172 orthologous DEGs were identified and they were grouped in 16 clusters (defined by a four-letter code) based on the upregulation or downregulation in tagatose-incubated samples compared to control samples in the two *Phytophthora* spp. and time points, such as modulation in *P. infestans* at 4 DAI (first letter); *P. infestans* at 10 DAI (second letter); *P. cinnamomi* at 4 DAI (third letter); *P. cinnamomi* at 10 DAI (fourth letter; Supplementary Table 11).

The RNA-Seq results were validated by qPCR analysis of 14 *Phytophthora* spp. genes (Supplementary Table 2) that were selected according to their expression profiles (six *P. infestans*, six *P. cinnamomi* and two orthologous genes belonging to different clusters) and functional categories (e.g., sugar metabolism, oxidative stress, and transport). A close correlation (Pearson *r* = 0.90) between RNA-Seq and qPCR expression data was observed (Supplementary Figure 14) and expression profiles agreed completely for 12 genes and differed slightly for two genes (Supplementary Table 2).

### Tagatose Incubation Causes a Severe Transcriptional Response in *Phytophthora infestans*

**FIGURE 3:**
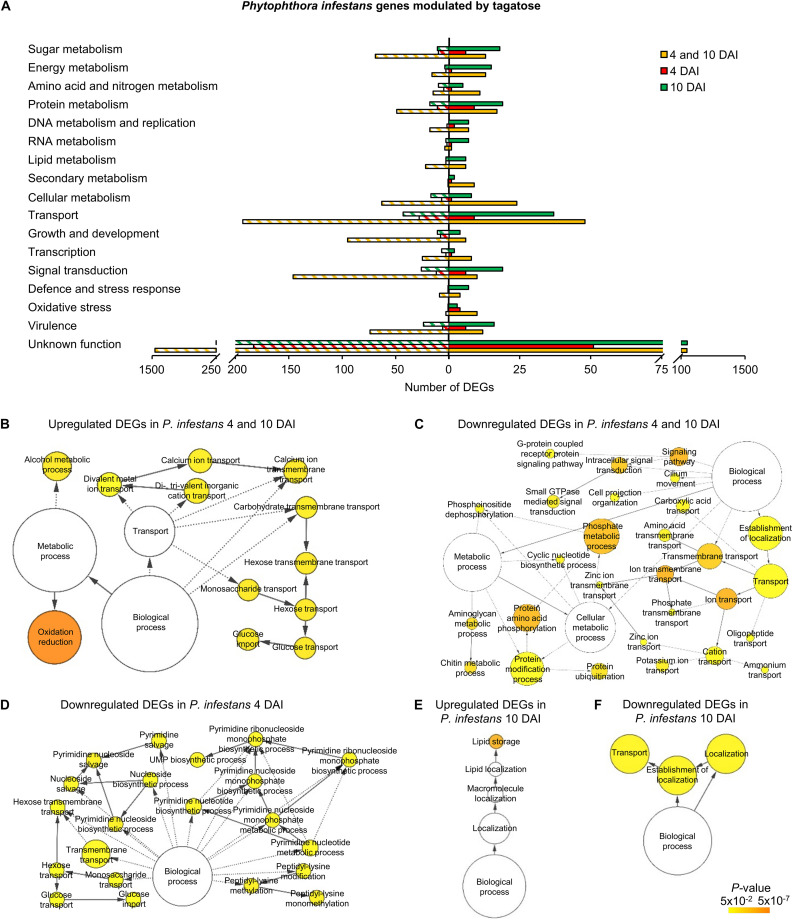
Functional annotation of genes modulated by tagatose in *P. infestans*. Differentially expressed genes (DEGs) were identified in *P. infestans* at 4 and 10 days after incubation (DAI) on pea agar medium in the presence of tagatose compared to the respective control samples grown in the absence of tagatose. Functional categories **(A)** were assigned based on the protein description of upregulated (solid bars) or downregulated (stripped bars) genes at both time points (4 and 10 DAI; orange) and exclusively at 4 DAI (red) or at 10 DAI (green; Supplementary Table 9). Biological networks of significantly enriched (*P* ≤ 0.05) Gene Ontology (GO) terms of *P. infestans* genes upregulated at 4 and 10 DAI **(B)**, downregulated at 4 and 10 DAI **(C)**, downregulated at 4 DAI **(D)**, upregulated at 10 DAI **(E)** and downregulated at 10 DAI **(F)** were identified using the BiNGO tool and visualized with Cytoscape software. The color scale legend indicates the level of significance for enriched GO terms and white nodes indicate not significantly overrepresented categories. Dotted lines indicate connection between biological process categories in the GO chart, where ancestor and child are omitted for simplicity. No significant GO enrichment was found for *P. infestans* genes upregulated at 4 DAI.

A high number of *P. infestans* genes was modulated at 4 and 10 DAI (2,688 DEGs: 427 upregulated and 2,247 downregulated; Figure 2A, Supplementary Figure 13A and Supplementary Table 9). The functional annotation revealed that upregulated genes of the 4 and 10 DAI cluster were mainly involved in primary metabolism (e.g., one epoxide hydrolase and two aldehyde dehydrogenases), protein metabolism, transport and energy metabolism (e.g., a fructose-bisphosphate aldolase, three glyceraldehyde-3-phosphate dehydrogenases and two phosphoglycerate mutases; Figure 3A and Supplementary Table 9). In particular, the GO biological process enrichment analysis revealed the overrepresentation of oxidation reduction category (e.g., four glutathione *S*-transferases and two quinone oxidoreductases) and transport-related processes [e.g., nine ATP-binding cassette (ABC) proteins and seven P-type ATPases (P-ATPase)] in the cluster of upregulated genes at 4 and 10 DAI (Figure 3B and Supplementary Table 9). *Phytophthora infestans* genes downregulated by tagatose at 4 and 10 DAI were mainly implicated in transport (e.g., one glucose transporter, three mitochondrial carriers and three amino acid transporters), signal transduction (e.g., three protein phosphatases and three cyclin-dependent kinases), growth and development (e.g., five myosin-like proteins, five dynein light chains and 21 kinesin-like proteins), sugar metabolism (e.g., an alpha-trehalose-phosphate synthase, a glucokinase, two lysosomal β-glucosidases, a β-glucosidase, an endo-1,4-β-xylanase and 18 glycoside hydrolases) and virulence (Figure 3A and Supplementary Table 9). Thus, the GO categories related to signaling and transport processes were overrepresented in the cluster of downregulated genes at 4 and 10 DAI (Figure 3C).

*Phytophthora infestans* genes modulated at 4 DAI (4 DAI cluster) and at 10 DAI (10 DAI cluster) included 385 DEGs (99 upregulated and 286 downregulated) and 842 DEGs (411 upregulated and 431 downregulated), respectively (Supplementary Figure 13 and Supplementary Table 9). Downregulated genes at 4 DAI were mainly involved in transport (e.g., two glucose transporters and two sugar transporters), signal transduction (e.g., two protein kinases) and protein metabolism (e.g., two serine protease inhibitors and three serine proteases; Figure 3A and Supplementary Table 9). Moreover, the GO categories related to transport (e.g., glucose transport) and pyrimidine metabolism were overrepresented in the cluster of downregulated genes at 4 DAI (Figure 3D and Supplementary Table 9). Upregulated genes of the 10 DAI cluster were mainly related to protein metabolism (Figure 3A) with the overrepresentation of the lipid storage GO category (Figure 3E and Supplementary Table 9). Moreover, downregulated genes at 10 DAI were mainly involved in transport (e.g., 10 ABC proteins and three mitochondrial carriers), virulence (six crinkler family proteins, one elicitin-like protein and one cutinase) and signal transduction (Figures 3A,F and Supplementary Table 9). However, a large fraction of genes with an unknown function was modulated in the 4 and 10 DAI (228 upregulated and 1,445 downregulated), 4 DAI (51 upregulated and 183 downregulated) and 10 DAI (236 upregulated and 252 downregulated) cluster, and indicated that that several yet-to-be identified proteins may have been involved in the *P. infestans* response to tagatose.

**FIGURE 4:**
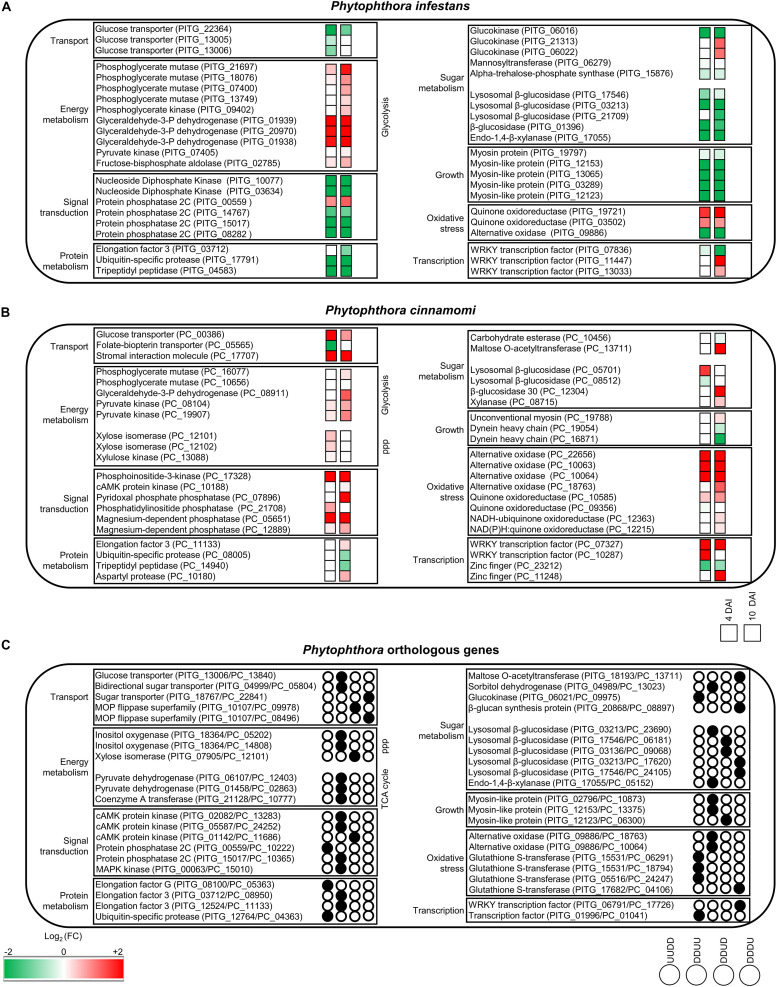
Overview of the main processes modulated by tagatose in *Phytophthor*a spp. Main cellular pathways affected by tagatose were identified for differentially expressed genes (DEGs) of *P. infestans* **(A)**, *P. cinnamomi* **(B)**, and *Phytophthora* spp. orthologous **(C)** genes at 4 and 10 days after incubation (DAI) on pea agar medium. For each *P. infestans* and *P. cinnamomi* gene, two squares represent the Log_2_-transformed fold change values of tagatose-incubated samples calculated as compared to control samples at 4 (left square) and 10 (right square) DAI, according to the color scale reported. Orthologous DEGs were grouped in 16 clusters (defined by a four-letter code, black circles) based on their upregulation (U) or downregulation (D) in tagatose-incubated samples [*P. infestans* at 4 DAI (first letter); *P. infestans* at 10 DAI (second letter); *P. cinnamomi* at 4 DAI (third letter); *P. cinnamomi* at 10 DAI (fourth letter)], such as genes upregulated in *P. infestans* and downregulated in *P. cinnamomi* at 4 DAI and at 10 DAI (UUDD), genes downregulated in *P. infestans* at 4 DAI and at 10 DAI and upregulated in *P. cinnamomi* at 4 DAI and at 10 DAI (DDUU), at 4 DAI (DDUD) or at 10 DAI (DDDU).

In summary, *P. infestans* response to tagatose incubation involved the upregulation of energy metabolism and oxidative stress response. However, the *P. infestans* transcriptional reprogramming was characterized by the downregulation of genes of transport-, sugar metabolism-, signal transduction- and growth-related processes (Figure 4A), in agreement with the inhibition of mycelial growth and alteration of sugar and amino acid content.

### Tagatose Incubation Causes an Efficient Transcriptional Response in *Phytophthora cinnamomi*

**FIGURE 5:**
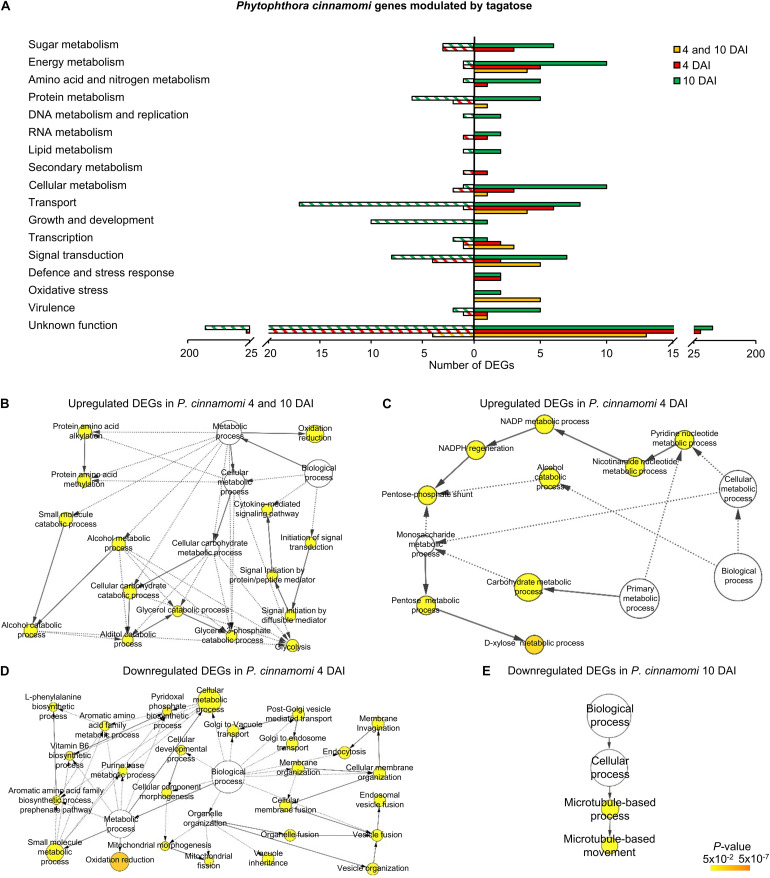
Functional annotation of genes modulated by tagatose in *Phytophthora cinnamomi*. Differentially expressed genes (DEGs) were identified in *P. cinnamomi* at 4 and 10 days after incubation (DAI) on pea agar medium in the presence of tagatose compared to the respective control samples grown in the absence of tagatose. Functional categories **(A)** were assigned based on the protein description of upregulated (solid bars) or downregulated (stripped bars) genes at both time points (4 and 10 DAI; orange) and exclusively at 4 DAI (red) or at 10 DAI (green; Supplementary Table 10). Biological networks of significantly enriched (*P* ≤ 0.05) Gene Ontology (GO) terms of *P. cinnamomi* genes upregulated at 4 and 10 DAI **(B)**, upregulated at 4 DAI **(C)**, downregulated at 4 DAI **(D)** and downregulated at 10 DAI **(E)** were identified using the BiNGO tool and visualized with Cytoscape software. The color scale legend indicates the level of significance for enriched GO terms and white nodes indicate not significantly overrepresented categories. Dotted lines indicate connection between biological process categories in the GO chart, where ancestor and child are omitted for simplicity. No significant GO enrichment was found for *P. cinnamomi* genes downregulated at 4 and 10 DAI and upregulated at 10 DAI.

The three clusters of *P. cinnamomi* DEGs included 41 genes modulated at 4 and 10 DAI (36 upregulated and 5 downregulated), 122 genes modulated at 4 DAI (71 upregulated and 51 downregulated) and 349 genes modulated at 10 DAI (146 upregulated and 203 downregulated; Figure 2B, Supplementary Figure 13B, and Supplementary Table 10). Although a large fraction of genes with an unknown function was found for *P. cinnamomi* DEGs (17, 78, and 228 in the 4 and 10 DAI, 4 and 10 DAI cluster, respectively), functional annotations revealed the upregulation of genes involved in signal transduction (e.g., one phosphoinositide-3-kinase and two magnesium-dependent phosphatases), oxidative stress (e.g., three alternative oxidases and one quinone oxidoreductase), energy metabolism (e.g., two pyruvate kinases and one glycerol-3-phosphate dehydrogenase) and transport (e.g., one stromal interaction molecule, one glucose transporter and one voltage-gated potassium channel) in the 4 and 10 DAI cluster (Figure 5A and Supplementary Table 10). As a consequence, the GO categories related to energy metabolism (e.g., glycerol 3-phosphate catabolic process and glycolosis), oxidation reduction and signaling were overrepresented in the cluster of upregulated genes at 4 and 10 DAI (Figure 5B and Supplementary Table 10).

Genes upregulated by tagatose at 4 DAI were mainly involved in transport (e.g., two aquaporins, one MtN3-like protein and one carboxylic acid transporter) and energy metabolism (two xylose isomerases, one xylulose kinase and one succinate dehydrogenase; Figure 5A and Supplementary Table 10) with the overrepresentation of energy-related GO categories (e.g., NADPH regeneration, pentose metabolic process and carbohydrate metabolic process; Figure 5C and Supplementary Table 10). On the other hand, downregulated genes at 4 DAI were mainly involved in signal transduction (e.g., two protein kinase and one protein phosphatase) with the overrepresentation of oxidative reduction and vesicle-mediated transport (Figures 5A,D and Supplementary Table 10). Upregulated genes of the 10 DAI cluster were mainly related to energy metabolism (e.g., two phosphoglycerate mutases, one glyceraldehyde-3-phosphate dehydrogenase and one pyruvate dehydrogenase; Figure 5A and Supplementary Table 10). Moreover, downregulated genes at 10 DAI were mainly involved in transport, growth and development (Figure 5A), as well as the GO categories of microtubule-based processes (Figure 5E and Supplementary Table 10). Thus, *P. cinnamomi* response to tagatose was characterized by the upregulation of genes implicated in transport-, energy metabolism- and oxidative stress-related processes (Figure 4B), possibly to adapt the cellular metabolism and minimize the alteration of the sugar content and mycelial growth.

### Tagatose Differentially Modulates Orthologous Genes in *Phytophthora infestans* and *P. cinnamomi*

**FIGURE 6:**
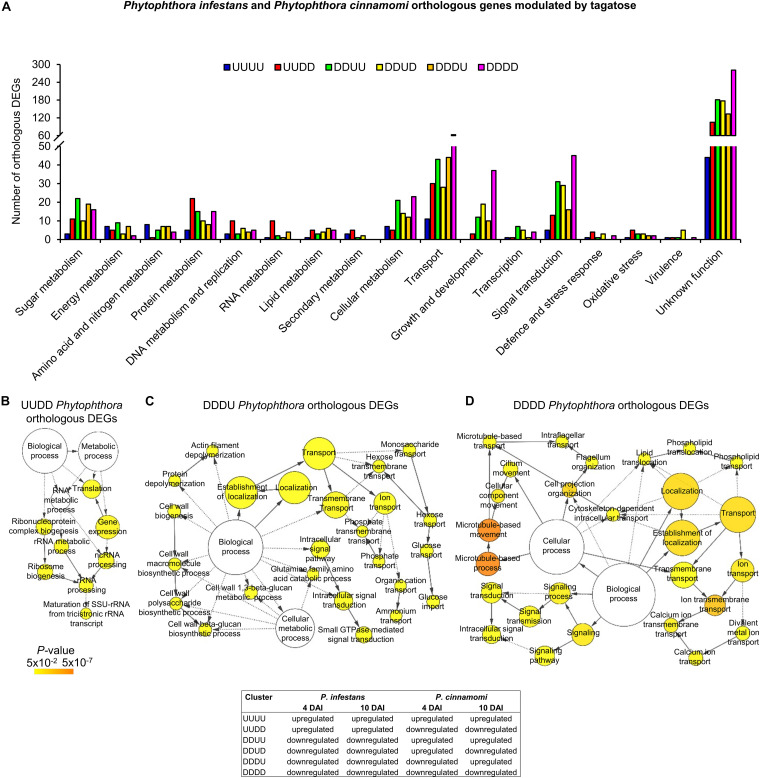
Functional annotation of *P. infestans and P. cinnamomi* orthologous genes modulated by tagatose. Orthologous genes of *P. infestans* and *P. cinnamomi* differentially expressed (orthologous DEGs) were identified at 4 and 10 days after incubation (DAI) on pea agar medium in the presence of tagatose compared to the respective control samples grown in the absence of tagatose. Orthologous DEGs were grouped in 16 clusters (defined by a four-letter code) based on their upregulation (U) or downregulation (D) in tagatose-incubated samples (Supplementary Table 11): *P. infestans* at 4 DAI (first letter); *P. infestans* at 10 DAI (second letter); *P. cinnamomi* at 4 DAI (third letter); *P. cinnamomi* at 10 DAI (fourth letter); such as the cluster of genes upregulated in *P. infestans* and downregulated in *P. cinnamomi* at 4 and 10 DAI (UUDD). Functional categories **(A)** of the clusters UUUU (blue), UUDD (red), DDUU (green), DDUD (yellow), DDDU (orange) and DDDD (pink) were assigned based on the protein description. Biological networks of significantly enriched (*P* ≤ 0.05) Gene Ontology (GO) terms of UUDD **(B)**, DDDU **(C)**, and DDDD **(D)** cluster of orthologous DEGs were identified using the BiNGO tool and visualized with Cytoscape software. The color scale legend indicates the level of significance for enriched GO terms and white nodes indicate not significantly overrepresented categories. Dotted lines indicate connection between biological process categories in the GO chart, where ancestor and child are omitted for simplicity. No significant GO enrichment was found for the UUUU, DDUU and DDUD clusters.

Although a large fraction of genes with an unknown function was found for the orthologous DEGs, functional annotations revealed that orthologous genes upregulated in both species and time points (UUUU cluster) were mainly implicated in transport (e.g., one mitochondrial carrier), amino acid and nitrogen metabolism (e.g., one glutamine amidotransferase, three aspartate aminotransferases and one histidinol-phosphate aminotransferase; Figure 6A and Supplementary Table 11). Orthologous genes upregulated in *P. infestans* and downregulated in *P. cinnamomi* (UUDD cluster) were mainly related to transport (e.g., six ABC proteins and one voltage-gated ion channel) and protein metabolism (e.g., seven ribosomal genes, one ubiquitin-specific protease and one elongation factor; Figure 6A and Supplementary Table 11) with the overrepresentation of translation and rRNA metabolic processes (Figure 6B). Moreover, orthologous genes downregulated in *P. infestans* and upregulated in *P. cinnamomi* (DDUU cluster) were mainly associated with sugar metabolism (e.g., seven glycoside hydrolases, one UDP-sugar pyrophospharylase, one sorbitol dehydrogenase, one endo-1,4-β-xylanase and one lysosomal β-glucosidase), transport (e.g., one glucose transporter, one bidirectional sugar transporter and one glycoside-cation symporter) and signal transduction (Figure 6A and Supplementary Table 11).

Orthologous genes of the DDUD cluster were mainly linked to transport [e.g., one multidrug/oligosaccharidyl-lipid/polysaccharide (MOP) flippase and two choline transporters), signal transduction, growth and development (e.g., four kinesin-like proteins, one dynein heavy chain and one myosin-like gene; Figure 6A and Supplementary Table 11). The DDDU cluster consisted of 273 orthologous DEGs that were mainly involved in transport (e.g., two phosphate transporters, a sugar transporter and a MOP flippase) and sugar metabolism (e.g., two lysosomal β-glucosidases, a maltose *O*-acetyltransferase and a β-glucan synthesis protein; Figure 6A and Supplementary Table 11) with the enrichment of monosaccharide transport and growth-related processes (e.g., actin filament depolymerization; Figure 6C and Supplementary Table 11). Orthologous genes downregulated in both species (DDDD cluster) were mainly related to transport, signal transduction, growth and development (Figure 6A and Supplementary Table 11) with the enrichment of the GO categories of transport, signaling and growth (e.g., microtubule-based process; Figure 6D and Supplementary Table 11). In summary, differential transcriptional regulation of *Phytophthora* spp. orthologous genes was found in presence of tagatose, including the upregulation of protein metabolism in *P. infestans* (UUDD cluster) and the upregulation of sugar metabolism, transport, signal transduction and growth-related processes in *P. cinnamomi* (DDUU, DDUD, and DDDU cluster; Figure 4C).

## Discussion

Tagatose showed nutritional or anti-nutritional properties for specific microbial taxa (Bautista et al., 2000; Perazzolli et al., 2020) and caused differential growth inhibition in some species belonging to the same genus, such as *Phytophthora* (Chahed et al., 2020). In particular, the tagatose dosage showing a differential effects on *Phytophthora* spp. growth was previously identified [5 g/L tagatose; (Chahed et al., 2020)] and it was used to assess metabolic and transcriptional changes. Differential response to tagatose incubation was found in *P. infestans* and *P. cinnamomi* with species-specific metabolic and transcriptional changes at the time points (4 and 10 DAI) that previously showed cellular ultrastructure changes in *P. infestans*, but not in *P. cinnamomi* (Chahed et al., 2020). In particular, sugar content and amino acid content were affected by tagatose in *P. infestans*, but not in *P. cinnamomi*. Moreover, a high number of genes was modulated by tagatose in *P. infestans* (3,915 DEGs) compared to *P. cinnamomi* (512 DEGs), suggesting that tagatose incubation caused a severe transcriptional reprogramming in *P. infestans*, rather than in *P. cinnamomi*. In addition, *P. infestans* DEGs were mainly repressed (75.70%), unlike *P. cinnamomi* (50.58%), suggesting global downregulation of functional processes associated with the growth inhibition caused by tagatose in *P. infestans*. Metabolic and transcriptional changes were assessed for each tagatose-incubated sample in comparison to the respective control sample, in order to detect the tagatose effects for each species and time point. However, the possible contribution of the different growth rate and developmental stage to the differential response of *P*. *infestans* and *P. cinnamomi* to tagatose cannot be totally excluded.

### Main Cellular Processes Affected by Tagatose Incubation in *Phytophthora infestans*

Tagatose incubation impaired *P. infestans* growth and caused severe impacts on sugar content and amino acid content with the downregulation of genes related to transport, sugar metabolism and growth-related process. In particular, tagatose incubation increased the content of fructose and decreased the content of glucose, mannose and ribose in *P. infestans*, but not in *P. cinnamomi*. Tagatose is the C-4 epimer of fructose and it may inhibit fructose-metabolizing enzymes in *P. infestans*, as previously reported for the fructokinase in mammalian (Lu et al., 2008) and in *Hyaloperonospora arabidopsidis* (Mochizuki et al., 2020), with the consequent increase of the fructose content. On the other hand, the decrease in glucose content could be ascribed to the tagatose-depended inhibition of *P. infestans* β-glucosidase enzymes (Corneo et al., 2021), which are involved in polysaccharide and disaccharide hydrolysis (Saha et al., 1994). As corroboration, genes encoding ß-glucosidase enzymes were mainly down- and up-regulated by tagatose in *P. infestans* and *P. cinnamomi*, respectively. In agreement with these findings, previous transcriptomic studies showed that the growth inhibition of *Phytophthora* spp., caused by biological products (melatonin), biocontrol bacteria (*L. capsici* AZ78) and fungicides (dimethomorph and metalaxyl), was associated with the downregulation of genes related to sugar metabolism (Tomada et al., 2017; Zhang et al., 2017; Hao et al., 2019) and to the decrease in sugar content (Maridueña-Zavala et al., 2017), indicating a strong correlation between sugar metabolism and *P. infestans* growth. Likewise, sugar- and growth-related genes involved in cell wall integrity and biogenesis (e.g., trehalose synthase, glucosyltransferase, glucose 4,6-dehydratases and β-glucan synthase genes) were downregulated by tagatose in *P. infestans*, but not in *P. cinnamomi*. The impact of tagatose on oomycete cell wall was previously reported in *H. arabidopsidis*, where tagatose inhibited the metabolism of mannan (Mochizuki et al., 2020), which is an essential component of oomycete cell wall (Melida et al., 2013). The alteration of cell wall integrity can negatively affect *P. infestans* pathogenicity (Resjö et al., 2017), and genes related to virulence and infection processes, such as RxLR (Kamoun, 2006), necrosis-inducing protein (NPP1) (Qutob et al., 2002) and thrombospondin (Robold and Hardham, 2005) were downregulated in tagatose-incubated *P. infestans*. Although possible alterations on pathogenicity should be validated by artificial inoculations on tomato plants, transcriptional changes indicated severe impacts of tagatose on key functional processes of *P. infestans*.

As a possible consequence of sugar metabolism inhibition, tagatose incubation decreased amino acid content in *P. infestans*, but not in *P. cinnamomi*. Previous studies showed that some fungal species (e.g., *Candida albicans*) can exploit amino acids as a carbon source under sugar-limiting conditions (Ene et al., 2014), suggesting that *P. infestans* could catabolise amino acids in the presence of tagatose. The amino acid catabolism involves cytoplasmic transaminases to form glutamic acid (Judelson et al., 2009), which is the only amino acid to increase in tagatose-incubated *P. infestans*. As corroboration, genes involved in glutamic acid biosynthesis (e.g., glutamine amidotransferase and histidinol-phosphate transaminase genes) were upregulated by tagatose, suggesting that amino acid metabolism was reprogrammed in *P. infestans* possibly to compensate the inhibition of sugar metabolism. Likewise, the growth inhibition of *Phytophthora* spp. caused by biological products (melatonin) and fungicides (e.g., ethylicin and SYP-14288) was previously associated with the inhibition of amino acid metabolism and protein synthesis (Zhang et al., 2017, 2020; Cai et al., 2019). For example, arginine content was decreased in tagatose-incubated *P. infestans* and genes involved in arginine biosynthesis (e.g., arginino-succinate synthase) were downregulated. The decrease of amino acid content was associated also with the downregulation of amino acid transporters in tagatose-incubated *P. infestans*. Similarly, the downregulation of amino acid transporter genes was previously linked to *P. infestans* growth inhibition caused by *Pseudomonas fluorescens* LBUM223 (Roquigny et al., 2018), indicating strong correlations between sugar metabolism, amino acid metabolism, amino acid transport and *P. infestans* growth.

### Processes With Opposite Modulation in *Phytophthora infestans* and in *P. cinnamomi* After Tagatose Incubation

Opposite modulation of genes involved in transport, energy metabolism, growth-related process, and oxidative stress response were found in *P. infestans* and *P. cinnamomi*, indicating species-specific reaction to tagatose incubation. In particular, tagatose incubation led to the down- and up-regulation of genes encoding sugar transporters (e.g., glucose transporters, bidirectional sugar transporters, glycoside-pentoside transporters and multidrug/oligosaccharidyl-lipid/polysaccharide flippases) in *P. infestans* and *P. cinnamomi*, respectively. Clinical studies showed that tagatose may act by attenuating glucose absorption in the human intestine (Donner et al., 1999). Likewise, the rare sugar sorbose inhibited glucose transport in *Saccharomyces cerevisiae* leading to growth retardation (van Uden, 1967), suggesting that the growth inhibitory effects of rare sugars may be mediated by glucose transport inhibition. Thus, the upregulation of glucose and sugar transporters in *P. cinnamomi* could be an efficient cellular response to mitigate tagatose inhibitory effects on sugar transporters and to maintain sufficient glucose uptake and energy production. Likewise, genes implicated in the tricarboxylic acid (TCA) cycle (e.g., malate synthase, succinate-semialdehyde dehydrogenase, succinate dehydrogenase and pyruvate dehydrogenase) and mitochondrial respiration (e.g., ubiquinone biosynthesis protein COQ7, cytochrome b5 and, NADH dehydrogenase), were down- and up-regulated by tagatose incubation in *P. infestans* and *P. cinnamomi*, respectively. In agreement with these findings, tagatose incubation decreased the content of TCA cycle intermediates in *P. infestans* (such as malic acid and succinic acid) (Corneo et al., 2021) and impaired normal respiration and ATP synthesis in *P. infestans*, but not in *P. cinnamomi* (Chahed et al., 2020), indicating species-specific effects of tagatose on some pathways of energy metabolism. As a possible consequence of energy limitation, genes associated with growth and development (e.g., myosin-like protein and actin-like protein) were mainly down- and up-regulated by tagatose incubation in *P. infestans* and *P. cinnamomi*, respectively. Likewise, *Phytophthora capsici* growth inhibition by the fungicide benzothiazole was previously associated with the downregulation of growth-related genes, such as those encoding actin and ankyrin repeat-protein (Mei et al., 2019).

Although genes involved in oxidative stress response were upregulated by tagatose incubation in both *Phytophthora* spp., some genes belonging to this functional category showed species-specific profiles. For example, glutathione *S*-transferase genes were up- and down-regulated in *P. infestans* and *P. cinnamomi*, respectively (UUDD cluster), while alternative oxidase genes showed opposite profile (DDUU cluster). Glutathione *S*-transferase gene is a marker of oxidative stress and it was induced by biocontrol bacteria (*L. capsici* AZ78 and *P. fluorescens* LBUM223) in *P. infestans* (Tomada et al., 2017; Roquigny et al., 2018). Thus, expression profiles of glutathione *S*-transferase genes agreed with the stronger accumulation of reactive oxygen species (ROS) previously found in *P. infestans* compared to *P. cinnamomi* during tagatose incubation (Chahed et al., 2020). Conversely, the upregulation of alternative oxidase genes in *P. cinnamomi* rather than *P. infestans* suggest a key contribution of these enzymes in the ROS detoxification, as previously found for the oxidative stress-handling machinery of *Ustilago maydis* (Juarez et al., 2006). Alternative oxidases are also key enzymes of the alternative respiration pathways in phytopathogenic fungi (Tian et al., 2020) and their expression was upregulated by non-fermentable carbon sources (e.g., glycerol and ethanol) in *C. albicans* (Huh and Kang, 2001). Thus, *P. cinnamomi* may upregulate alternative oxidase pathways, in order to mitigate the oxidative stress and to allow alternative respiration, in agreement with the normal ROS level and oxygen consumption rate previously found during tagatose incubation (Chahed et al., 2020). Moreover, genes related to signal transduction were mainly down- and up-regulated in *P. infestans* (e.g., protein phosphatases 2C and cAMP kinases) and *P. cinnamomi*, respectively. Phosphatase 2C genes were downregulated by oxidative stress in *Nicotiana tabacum* (Vranová et al., 2000) and signaling genes (e.g., cAMP domain-containing protein) were downregulated by growth inhibition in *P. capsici* (Mei et al., 2019), suggesting the activation of complementary signal transduction pathways in *P. infestans* and *P. cinnamomi* to modulate the response to tagatose incubation.

### Main Cellular Processes Affected by Tagatose Incubation in *Phytophthora cinnamomi*

Mycelial growth, sugar and amino acid content were not affected by tagatose incubation in *P. cinnamomi* and the transcriptional response revealed the upregulation of genes related to transport, energy metabolism, sugar metabolism and oxidative stress. In particular, the response of *P. cinnamomi* included the upregulation of genes related to pentose metabolism, such as xylose isomerase, xylulose kinase and inositol oxygenase, indicating the upregulation of alternative sugar catabolism. Previous studies showed that blocking hexose entry into glycolysis upregulated pentose metabolism in *Aspergillus nidulans* (Khosravi et al., 2018) and that xylulose kinase co-expression with arabinan-degrading genes was essential for *Aspergillus niger* growth on xylose and arabinose (VanKuyk et al., 2001). Likewise, genes encoding sorbitol dehydrogenase and arabinan-degrading enzymes responsible for the release of pentose sugars from polysaccharides side chains (e.g., endo-1,4-beta-xylanase, arabinogalactan endo-beta-1,4-galactanase, arabinan endo-1,5-alpha-L-arabinosidase) were upregulated by tagatose incubation in *P. cinnamomi*. Consequently, the pentose sugars originated by these enzymatic activities can be assimilated into the pentose phosphate pathway, suggesting that *P. cinnamomi* metabolized alternative sugars and upregulated the pentose metabolism to alleviate metabolic impacts caused by tagatose. In addition, the *P. cinnamomi* response included the upregulation of a mannitol dehydrogenase gene, which was upregulated in fungicide-resistant *Phytophthora* isolates (Childers et al., 2015), and the upregulation of transcription factors (e.g., WRKY, zink finger proteins and MYB-like DNA-binding protein), suggesting the activation of an efficient machinery to modulate the response to tagatose incubation.

### Processes Commonly Affected by Tagatose Incubation in Both *Phytophthora* spp.

Some oxidative stress-related genes (e.g., quinone oxidoreductase) were upregulated in *P. infestans* and in *P. cinnamomi*, indicating that both species incurred an oxidative stress after tagatose incubation. However, the efficient scavenging of reactive oxygen species found in *P. cinnamomi* (Chahed et al., 2020) was associated with the upregulation of additional genes (such as alternative oxidases discussed above), which were downregulated in *P. infestans*. In both *Phytophthora* spp., some genes involved in the energy metabolism of glycolysis (e.g., phosphoglycerate mutase, glyceraldehyde-3-P dehydrogenase and pyruvate kinase) were upregulated by tagatose incubation. Tagatose was patented as glycolysis inhibitor in humans (Kim et al., 2014) and showed inhibitory effects on glycolysis-related enzymes in *H. arabidopsidis* (fructokinase and phosphomannose isomerase) (Mochizuki et al., 2020) and *Escherichia coli* (fructose phosphate aldolase) (Stellmacher et al., 2016). As a possible consequence of glycolysis inhibition, *P. infestans* and *P. cinnamomi* upregulated a pyruvate phosphate dikinase gene (cluster UUUU) to generate pyruvate, as a bypass of pyruvate kinase in glycolysis reactions (Judelson et al., 2009). Likewise, genes encoding glycerol-3-phosphate dehydrogenase were upregulated in both *Phytophthora* spp. and they can reinforce glycolysis pathways by providing an extra source of dihydroxyacetone phosphate (Lalle et al., 2015), as an attempted reaction to alleviate metabolic impacts derived from the glycolysis inhibition. The enzymatic inhibition caused by tagatose can be ascribed to the structural similarity with fructose and to the possible interference with the substrate binding by the active site, as in the case of the mammalian fructokinase (Lu et al., 2008) and *S. mutans* glucosyltransferase (Hasibul et al., 2018). This competitive effect was also suggested by the fructose-dependent attenuation of tagatose effects in *P. infestans* (Corneo et al., 2021) and in *S. mutants* (Hasibul et al., 2018), but further enzymatic studies are required in order to better clarify the inhibitory effects of tagatose on *Phytophthora* spp. enzymes. Likewise, metabolic and transcriptomic studies at early time points in presence of different dosages of rare sugars and/or common sugars will be required, in order to investigate the early response of *Phytophthora* spp. to tagatose and to clarify possible dose-dependent interactions between rare sugar and common sugar metabolism.

## Conclusion

The differential inhibitory effect of tagatose on *P. infestans* and *P. cinnamomi* was associated with species-specific metabolic and transcriptional changes. In particular, an attempted response was upregulated by *P. infestans*, but it was not sufficient to contrast the negative effects of tagatose incubation on mycelial growth, sugar content and amino acid content. Thus, *P. infestans* transcriptional reprogramming was mainly characterized by the severe downregulation of genes implicated in transport, sugar metabolism, signal transduction and growth-related process. Conversely, *P. cinnamomi* response to tagatose incubation was characterized by the activation of processes related to transport, energy metabolism, sugar metabolism and oxidative stress-related, in order to limit negative impacts on mycelial growth, sugar content and amino acid content. In particular, *P. cinnamomi* was able to implement multiple pathways to modulate the cellular metabolism based on the upregulation of genes related to glucose transport, pentose metabolism, TCA cycle, ROS detoxification, mitochondrial and alternative respiration. These metabolic and transcriptional results represent a major contribution to the characterization of the species-specific mode of action of tagatose on *Phytophthora* spp. and might pave the way for functional genomic and biochemical studies to further characterize enzymatic reactions affected by this rare sugar.

## Data Availability Statement

The sequences were deposited at the Sequence Read Archive of the National Center for Biotechnology under the BioProject number PRJNA622764. The datasets generated for this study can be found in online repositories. The names of the repository/repositories and accession number(s) can be found in the article/Supplementary Material.

## Author Contributions

AC and AN carried out the experiments, sample collection, and RNA extraction. VL carried out the qPCR experiments. MM analyzed the RNA-Seq data. PEC helped to carry out the experiments and sample collection. AC, MP, EAB, GP, and IP contributed to data interpretation and the manuscript writing. MP conceived the study, designed the experiments, and coordinated all research activities. All authors revised and approved the final manuscript.

## Conflict of Interest

AC and AN were employed by Biological Products for Agriculture (Bi-PA). The remaining authors declare that the research was conducted in the absence of any commercial or financial relationships that could be construed as a potential conflict of interest.

## Footnotes

1 https://protists.ensembl.org/Phytophthora_infestans/Info/Index

2 https://mycocosm.jgi.doe.gov/Phyci1/Phyci1.home.html

3 http://bioinformatics.psb.ugent.be/webtools/Venn/

4 www.python.org

5 http://www.uniprot.org

## Supplementary Material

Supplementary Figure 1 *Phytophthora* spp. growth.

Supplementary Figure 2 Effect of tagatose on *Phytophthora* spp. amino acid content.

Supplementary Figure 3 Correlation matrixes of *Phytophthora infestans* control samples at 4 days after incubation.

Supplementary Figure 4 Correlation matrixes of *P. infestans* control samples at 10 days after incubation.

Supplementary Figure 5 Correlation matrixes of *P. infestans* tagatose-incubated samples at 4 days after incubation.

Supplementary Figure 6 Correlation matrixes of *P. infestans* tagatose-incubated samples at 10 days after incubation.

Supplementary Figure 7 Correlation matrixes of *P. cinnamomi* control samples at 4 days after incubation.

Supplementary Figure 8 Correlation matrixes of *P. cinnamomi* control samples at 10 days after incubation.

Supplementary Figure 9 Correlation matrixes of *P. cinnamomi* tagatose-incubated samples at 4 days after incubation.

Supplementary Figure 10 Correlation matrixes of *P. cinnamomi* tagatose-incubated samples at 10 days after incubation.

Supplementary Figure 11 Principal component analysis (PCA) of *Phytophthora* spp. samples.

Supplementary Figure 12 Volcano Plot analysis for the identification of differentially expressed genes in *Phytophthora* spp.

Supplementary Figure 13 Venn diagrams of genes modulated by tagatose in *Phytophthora* spp.

Supplementary Figure 14 Correlation analysis of gene expression data assessed by RNA-Seq and quantitative real-time PCR.

Supplementary Table 1 Sugar content in the pea agar medium.

Supplementary Table 2 Primer sequences of the *Phytophthora* spp. genes analyzed by quantitative real-time PCR.

Supplementary Table 3 RNA-Seq sequencing and mapping results of each replicate.

Supplementary Table 4 Expression levels of *Phytophthora infestans* genes.

Supplementary Table 5 Expression levels of *P. cinnamomi* genes.

Supplementary Table 6 Pearson correlation values of *P. infestans* samples.

Supplementary Table 7 Pearson correlation values of *P. cinnamomi* samples.

Supplementary Table 8 Pearson correlation values of *Phytophthora* spp. samples.

Supplementary Table 9 Fold change values and functional annotation of genes modulated by tagatose in *P. infestans*.

Supplementary Table 10 Fold change values and functional annotation of genes modulated by tagatose in *P. cinnamomi*.

Supplementary Table 11 Expression profiles and functional annotation of *P. infestans* and *P. cinnamomi* orthologous genes modulated by tagatose.
