# Supplementary material for: The Differential Growth Inhibition of Phytophthora spp. Caused by the Rare Sugar Tagatose Is Associated With Species-Specific Metabolic and Transcriptional Changes
Source: Front Microbiol. 2021 Jul 7;12:711545. doi: 10.3389/fmicb.2021.711545 (PMC8292896; doi:10.3389/fmicb.2021.711545)
Supplement: Supplementary Figure 1 — Phytophthora spp. growth. [file Data_Sheet_1.pdf]

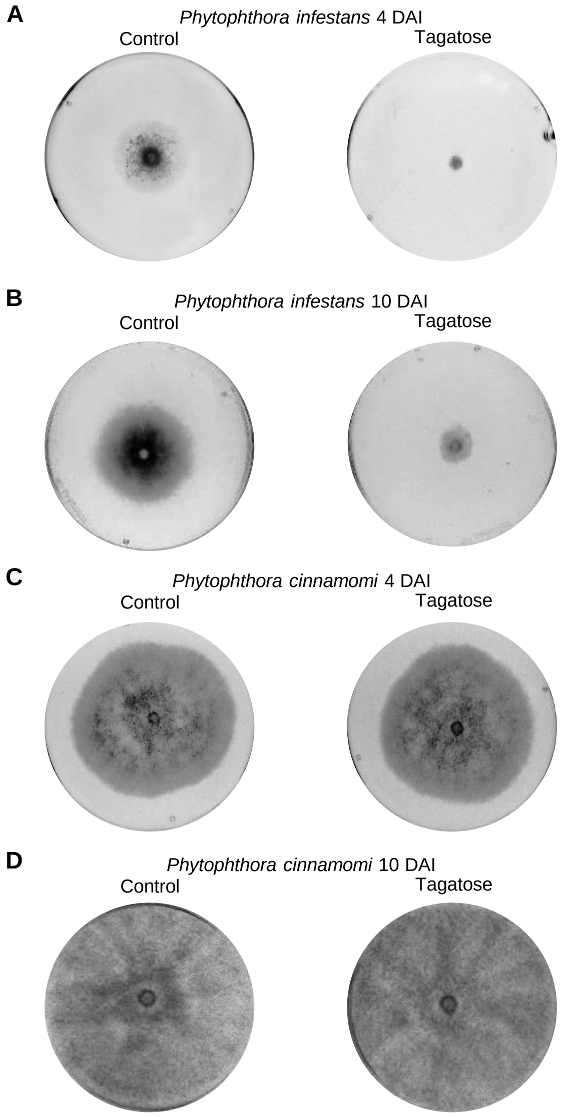

FIGURE S1 | *Phytophthora* spp. growth. Representative pictures of *P. infestans* **(A, B)** and *P. cinnamomi* **(C, D)** growth at four and ten days after incubation (DAI) on pea agar medium in the absence (control) and presence of 5 g/L tagatose.

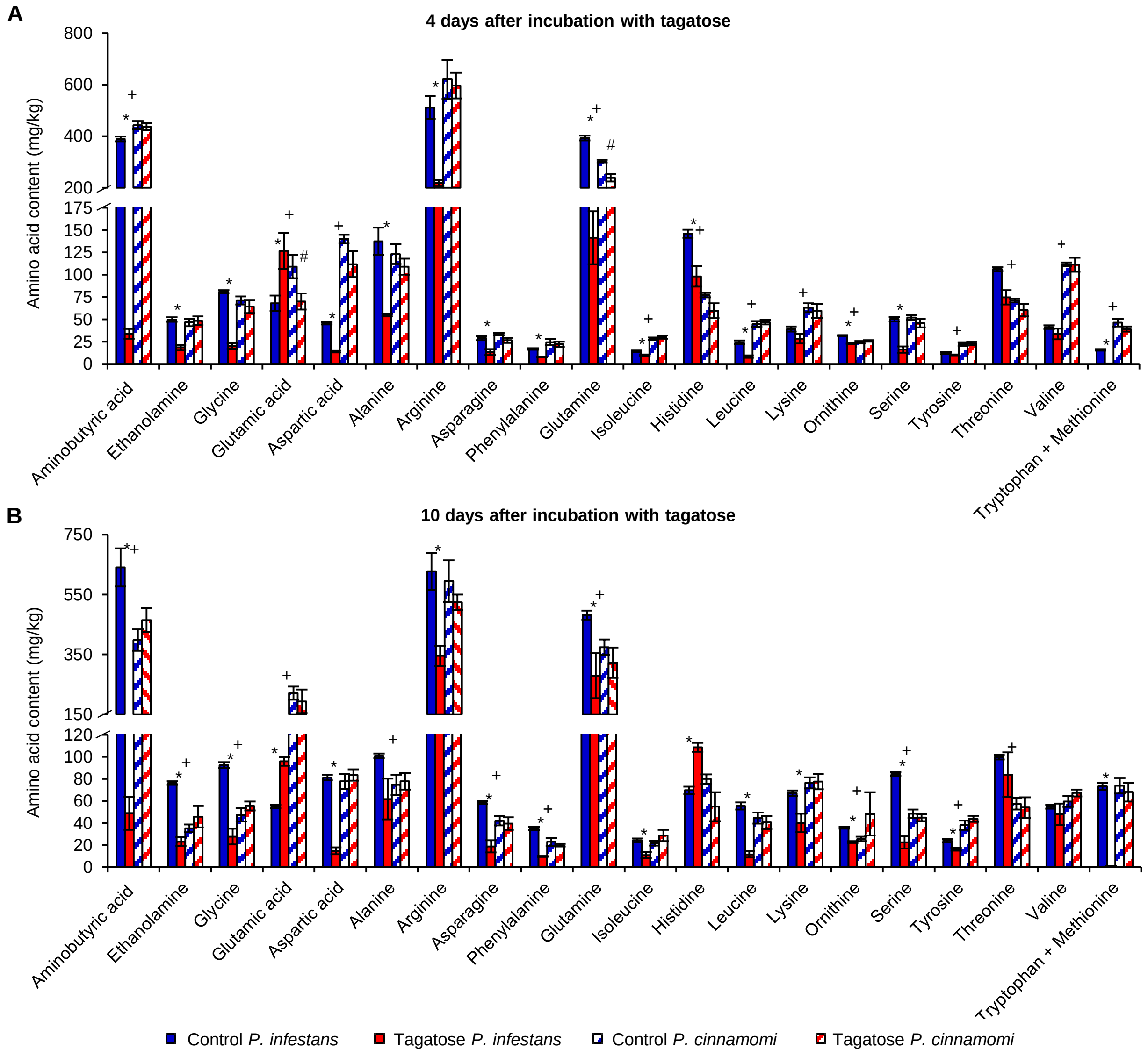

FIGURE S2 | Effect of tagatose on *Phytophthora* spp. amino acid content. *Phytophthora infestans* (solid bars) and *P. cinnamomi* (stripped bars) amino acid content was quantified at four **(A)** and ten **(B)** days after incubation (DAI) on pea agar medium in the absence (control, blue) and presence of tagatose (red). The two-way analysis of variance (two-way ANOVA) showed no significant differences between the two experimental repetitions ($P > 0.05$, three replicates per experiment) and data from the two experiments were pooled. Mean and standard error values of six replicates from the two experiments are presented for each treatment. For each amino acid and time point, significant differences between tagatose-incubated samples and control samples are marked with an asterisk (*) for *P. infestans* and a hashtag (#) for *P. cinnamomi*, according to the Student's t-test ($P \leq 0.05$). Significant differences between *P. infestans* control and *P. cinnamomi* control for each amino acid are marked with a plus sign (+), according to the Student's t-test ($P \leq 0.05$). Citrulline was not detected and omitted in the chart.

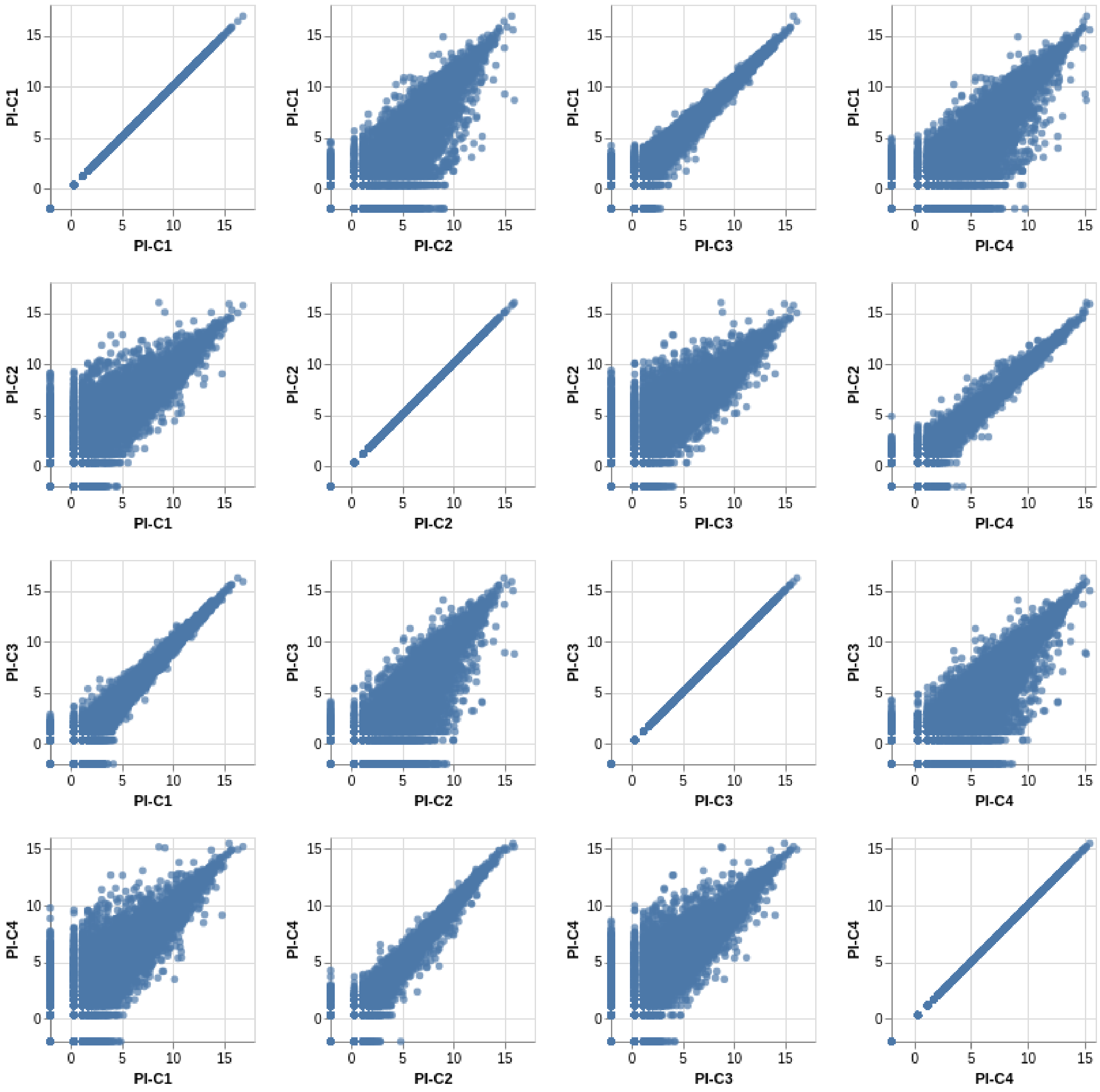

FIGURE S3 | Correlation matrixes of *Phytophthora infestans* control samples at four days after incubation. Pearson correlation analysis was carried out on transcriptomic data of *P. infestans* (PI) mycelium collected at four days after incubation (DAI) on pea agar medium in the absence (control, C) of tagatose in quadruplicate (numbers from 1 to 4).

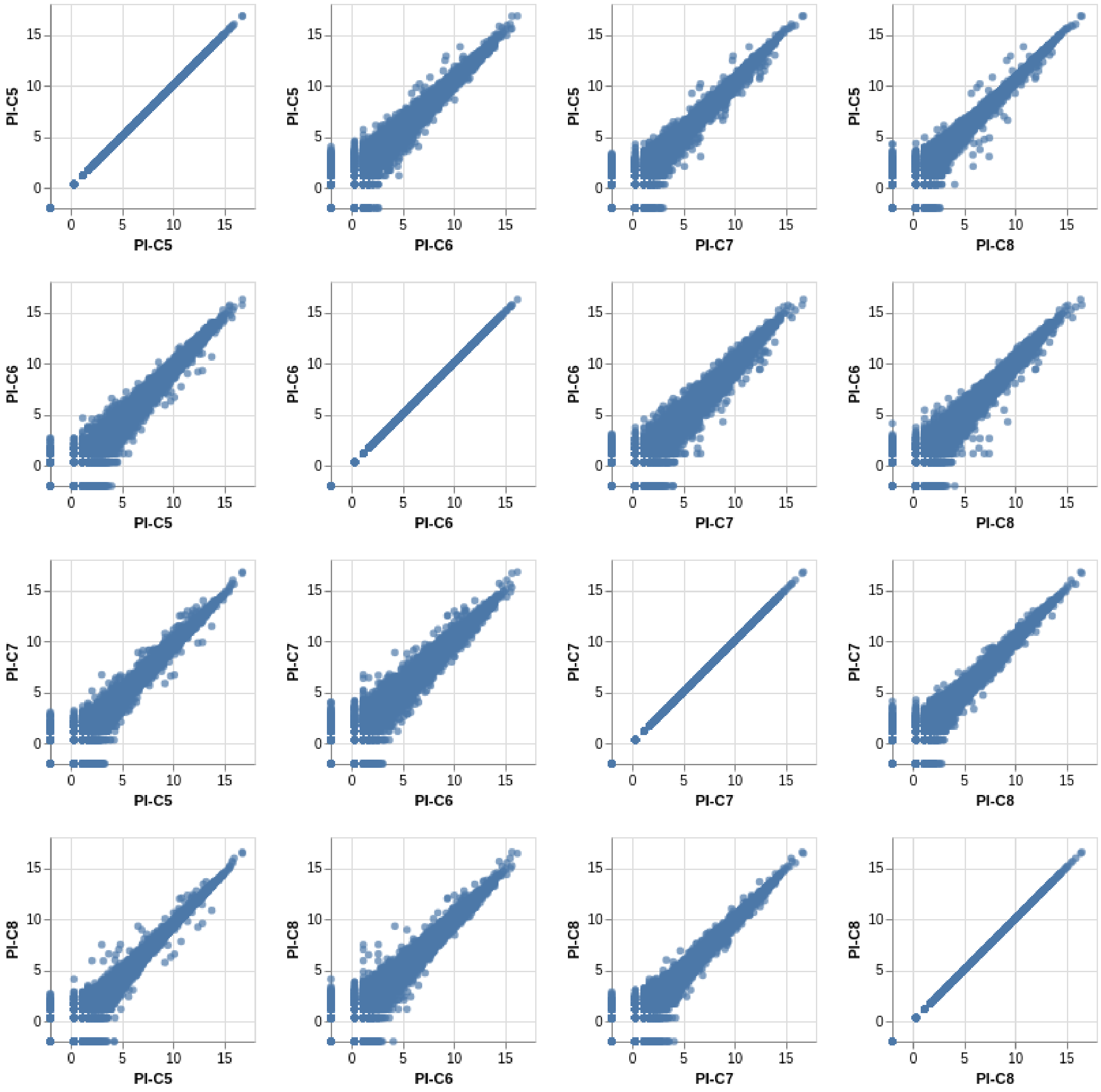

FIGURE S4 | Correlation matrixes of *Phytophthora infestans* control samples at ten days after incubation. Pearson correlation analysis was carried out on transcriptomic data *P. infestans* (PI) mycelium collected at ten days after incubation (DAI) on pea agar medium in the absence (control, C) of tagatose in quadruplicate (numbers from 5 to 8).

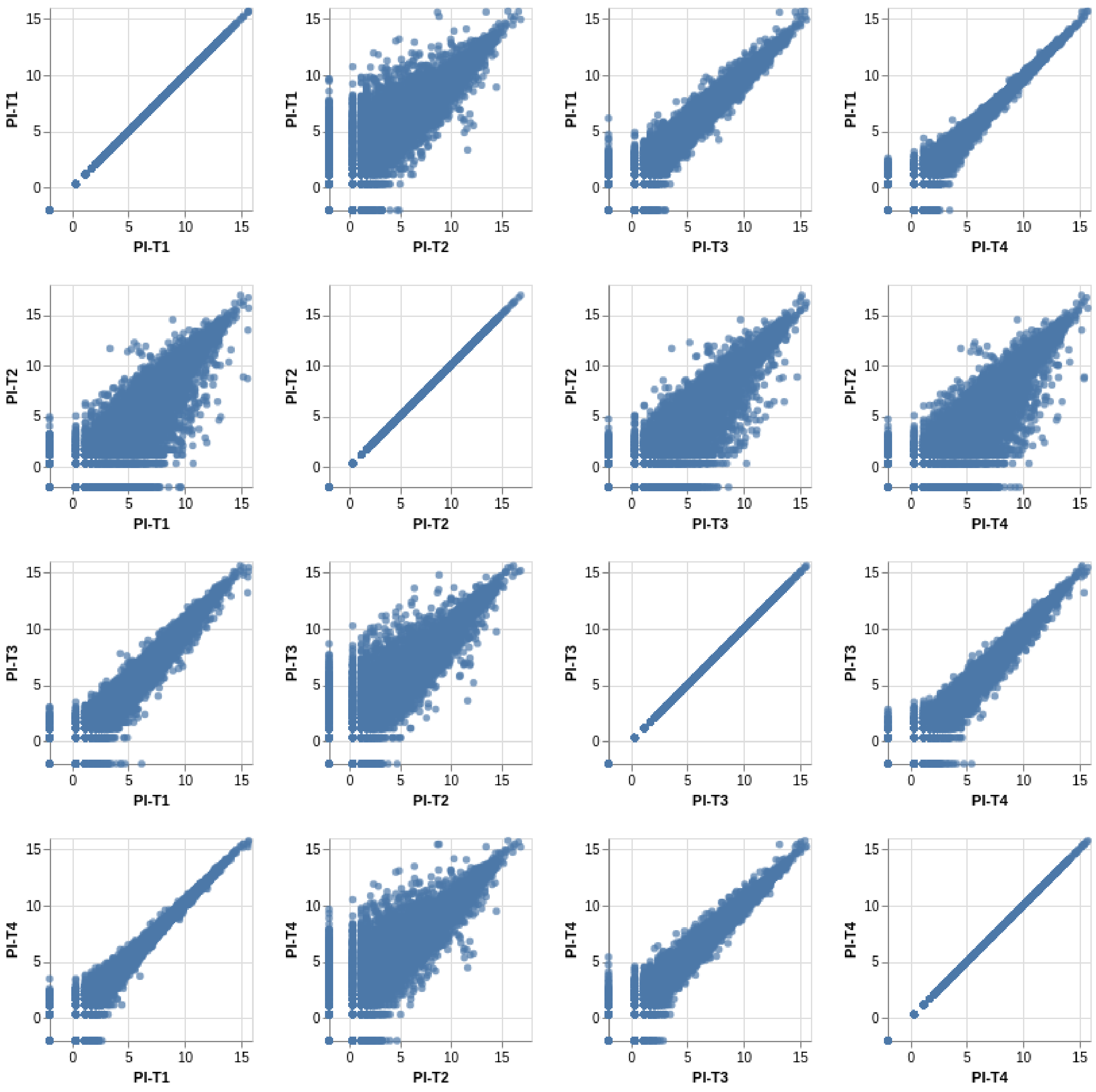

FIGURE S5 | Correlation matrixes of *Phytophthora infestans* tagatose-incubated samples at four days after incubation. Pearson correlation analysis was carried out on transcriptomic data of *P. infestans* (PI) mycelium collected at four days after incubation (DAI) on pea agar medium in the presence of tagatose (T) in quadruplicate (numbers from 1 to 4).

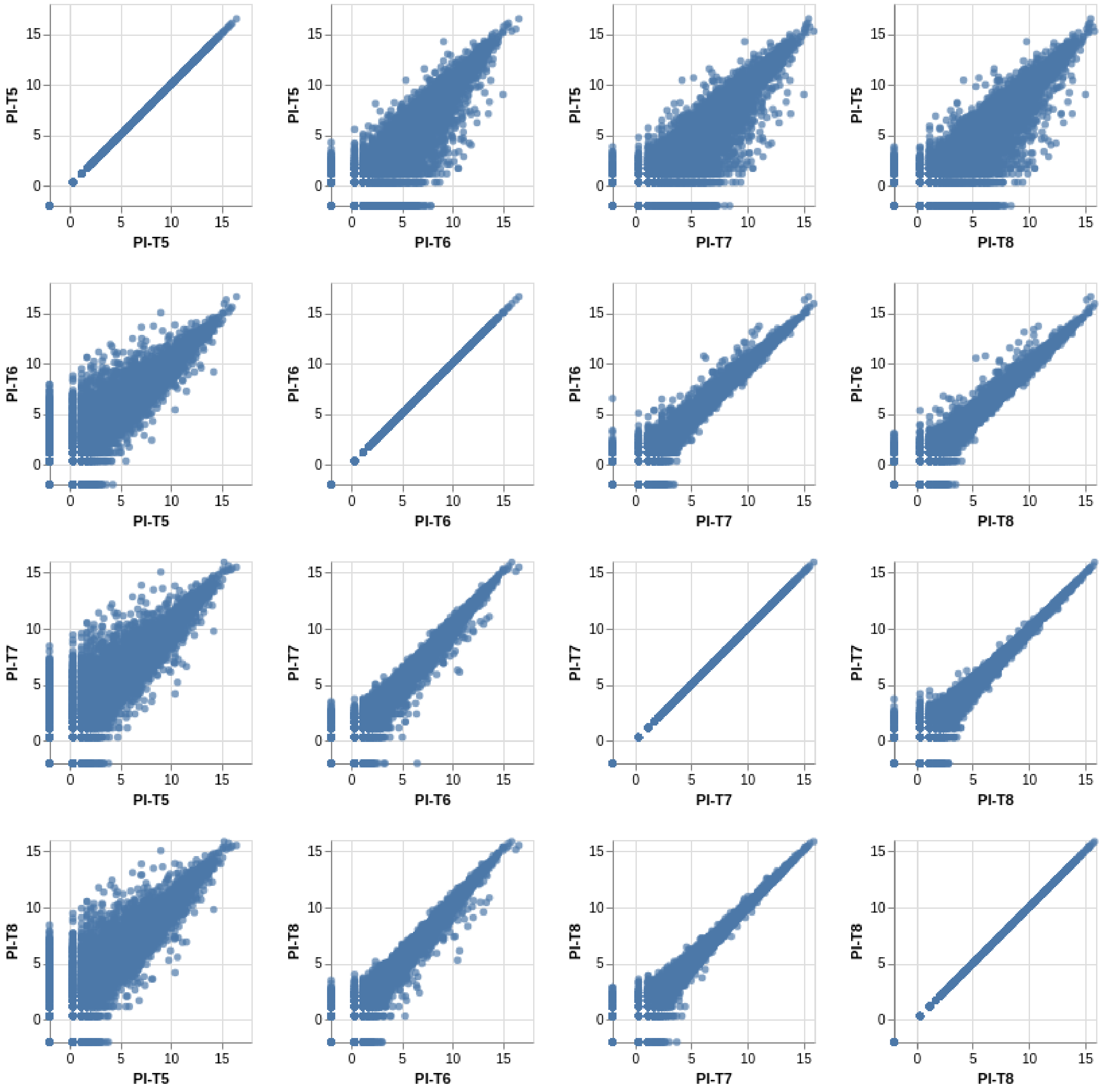

FIGURE S6 | Correlation matrixes of *Phytophthora infestans* tagatose-incubated samples at ten days after incubation. Pearson correlation analysis was carried out on transcriptomic data of *P. infestans* (PI) mycelium collected at ten days after incubation (DAI) on pea agar medium in the presence of tagatose (T) in quadruplicate (numbers from 5 to 8).

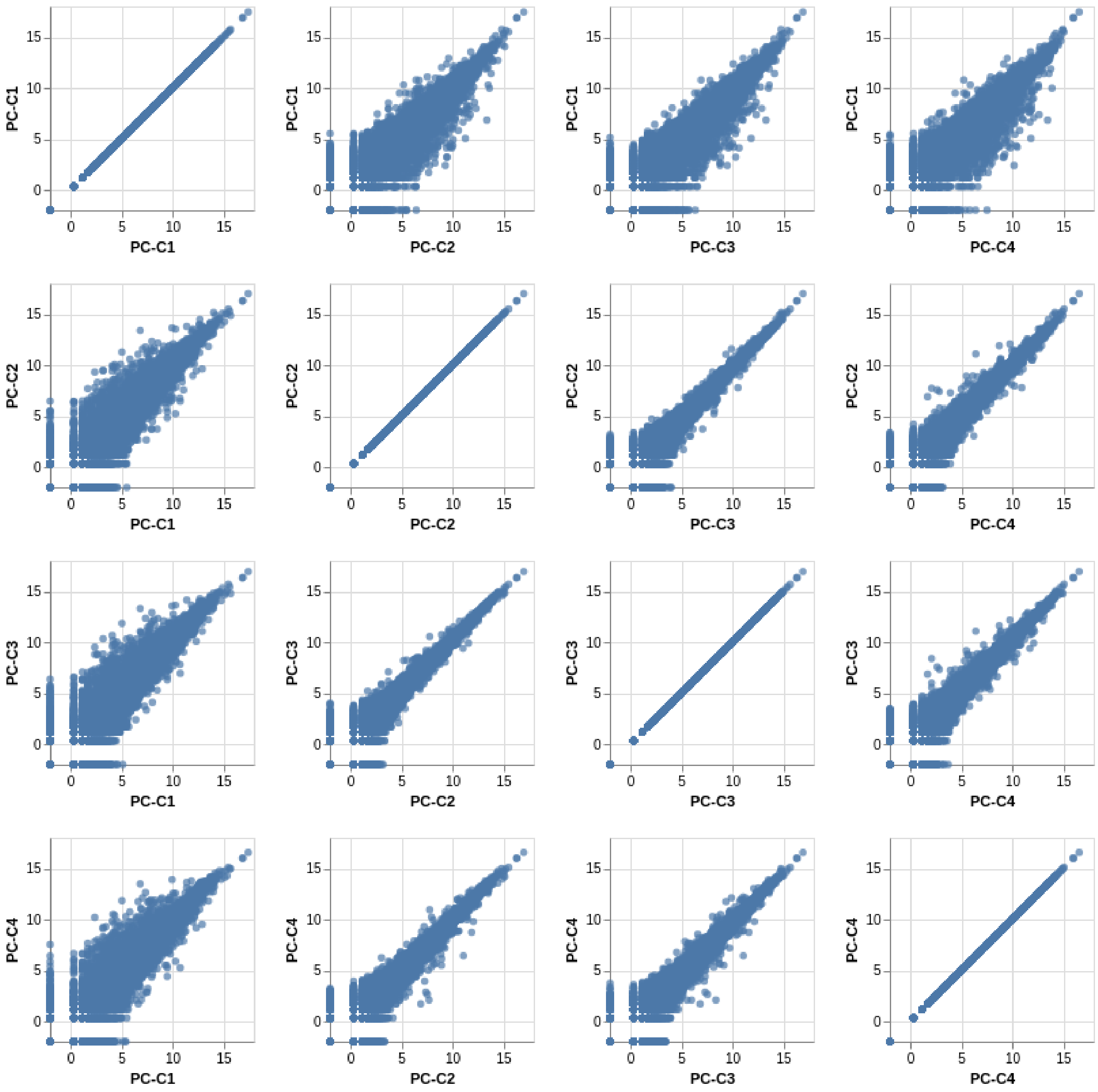

FIGURE S7 | Correlation matrixes of *Phytophthora cinnamomi* control samples at four days after incubation. Pearson correlation analysis was carried out on transcriptomic data of *P. cinnamomi* (PC) mycelium collected at four days after incubation (DAI) on pea agar medium in the absence (control, C) of tagatose in quadruplicate (numbers from 1 to 4).

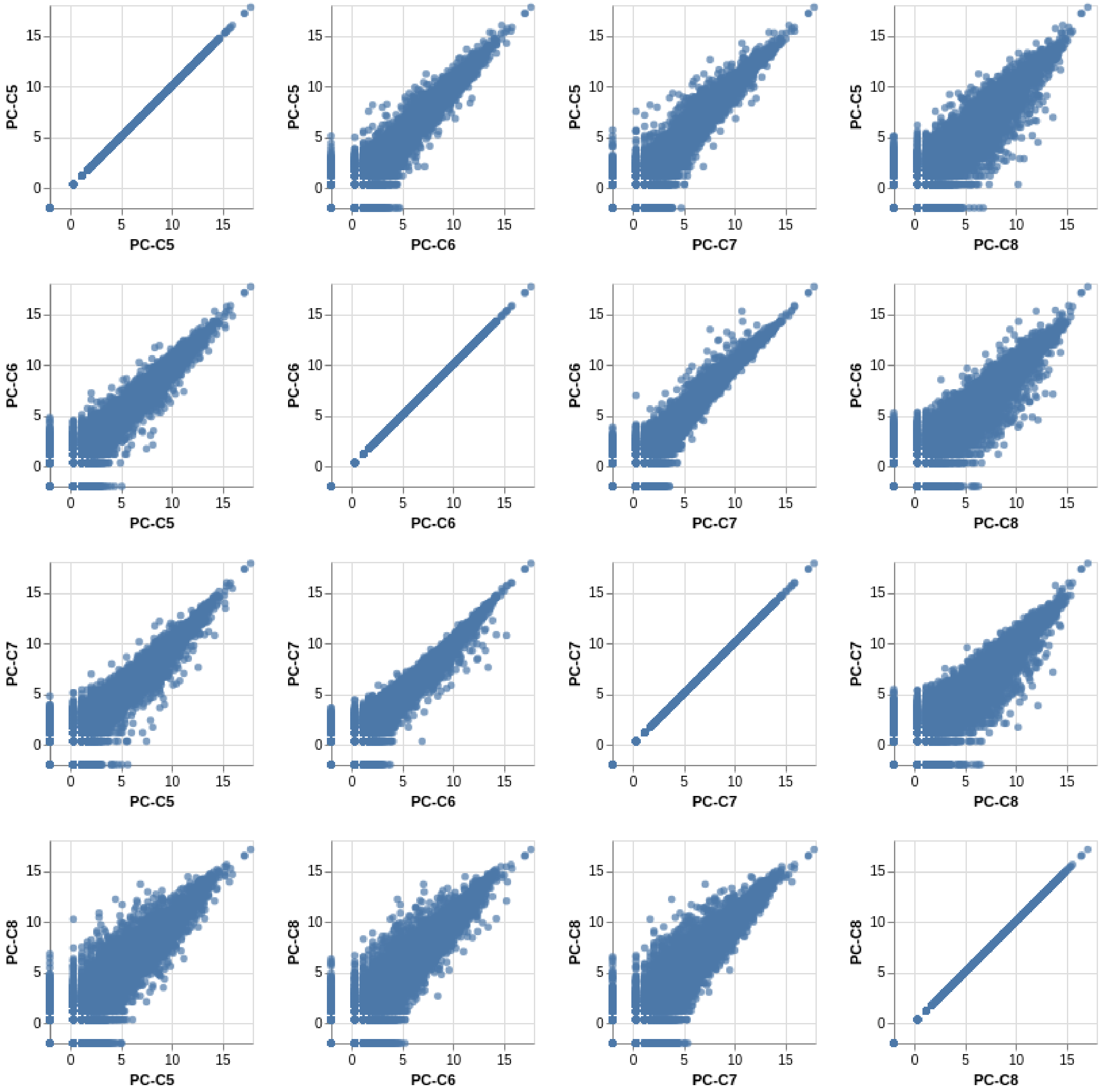

FIGURE S8 | Correlation matrixes of *Phytophthora cinnamomi* control samples at ten days after incubation. Pearson correlation analysis was carried out on transcriptomic data of *P. cinnamomi* (PC) mycelium collected at ten days after incubation (DAI) on pea agar medium in the absence (control, C) of tagatose in quadruplicate (numbers from 5 to 8).

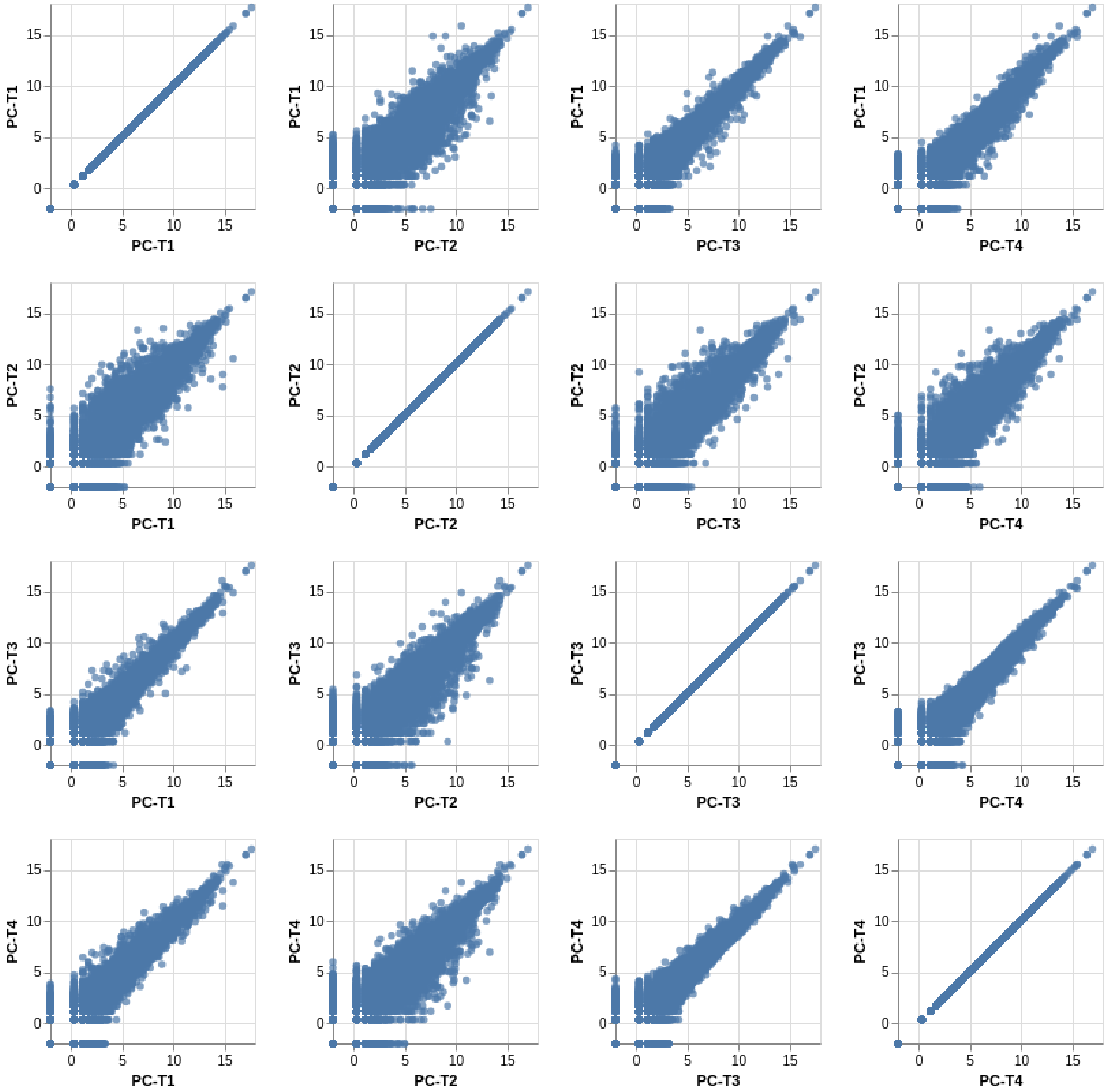

FIGURE S9 | Correlation matrixes of *Phytophthora cinnamomi* tagatose-incubated samples at four days after incubation. Pearson correlation analysis was carried out on transcriptomic data of *P. cinnamomi* (PC) mycelium collected at four days after incubation (DAI) on pea agar medium in the presence of tagatose (T) in quadruplicate (numbers from 1 to 4).

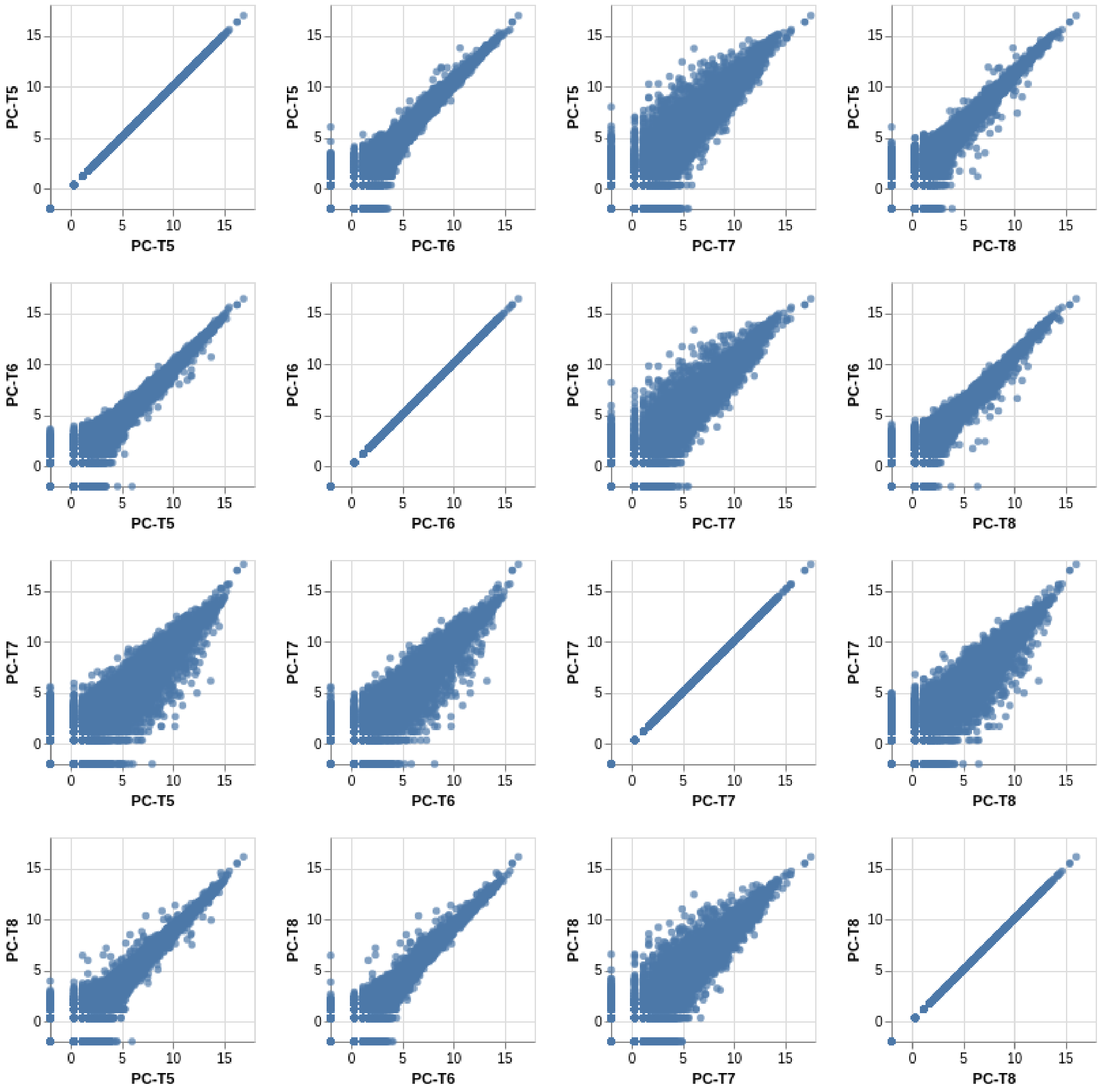

FIGURE S10 | Correlation matrixes of *Phytophthora cinnamomi* tagatose-incubated samples at ten days after incubation. Pearson correlation analysis was carried out on transcriptomic data of *P. cinnamomi* (PC) mycelium collected at ten days after incubation (DAI) on pea agar medium in the presence of tagatose (T) in quadruplicate (numbers from 8 to 8).

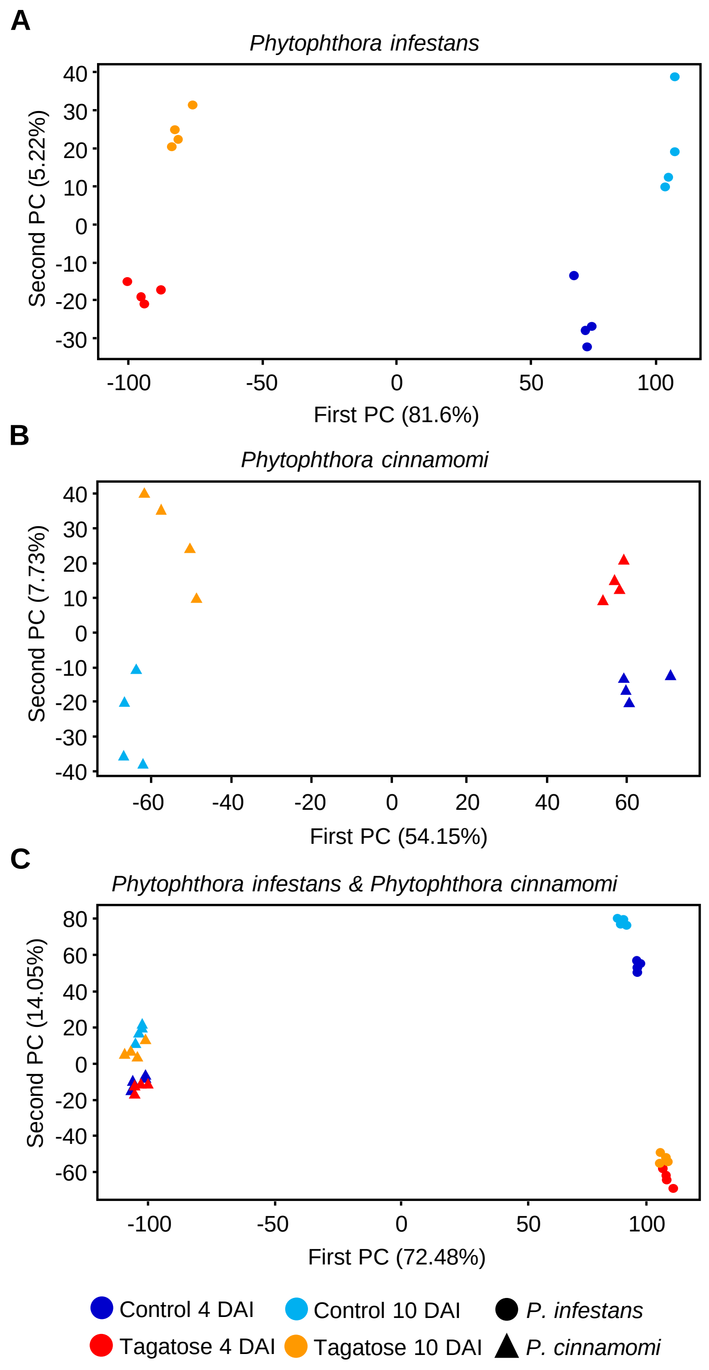

FIGURE S11 | Principal component analysis (PCA) of *Phytophthora* spp. samples. PCA of *Phytophthora infestans* **(A)**, *P. cinnamomi* **(B)** and *Phytophthora spp.* **(C)** samples was obtained on Limma-normalised expression values of *P. infestans* (circles) and *P. cinnamomi* (triangles) genes at four and ten days after incubation (DAI) on pea agar medium in the absence (control; blue, 4 DPI and light blue, 10 DAI) and presence of tagatose (red, 4 DPI and orange, 10 DAI) in four replicates. The percentage of variance explained by each principal component (PC) is reported in brackets.

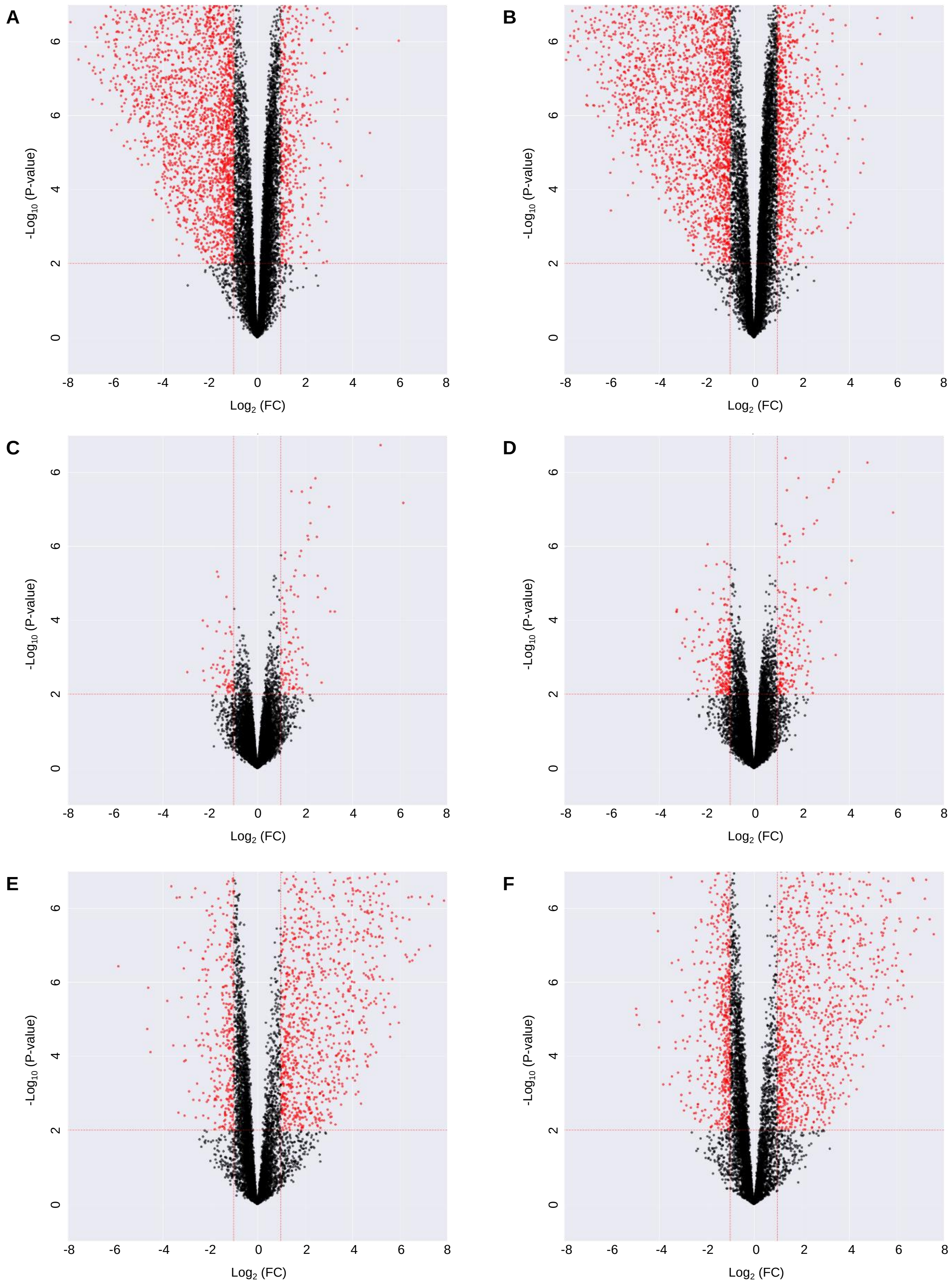

FIGURE S12 | Volcano Plot analysis for the identification of differentially expressed genes (DEGs) in *Phytophthora* spp. A double cutoff on *P*-value ($P \leq 0.01$) and minimum Log_2 fold change (FC) of one [Log_2 (FC) ≥ 1 or Log_2 (FC) ≤ -1] was used to select DEGs (red dots) in the following pairwise comparisons: tagatose-incubated vs. control of *Phytophthora infestans* at 4 DAI **(A)**, tagatose-incubated vs. control of *P. infestans* at 10 DAI **(B)**, tagatose-incubated vs. control of *P. cinnamomi* at 4 DAI **(C)**, tagatose-incubated vs. control of *P. cinnamomi* at 10 DAI **(D)**. *Phytophthora* orthologous DEGs were identified [$P \leq 0.01$; Log_2 (FC) ≥ 1 or Log_2 (FC) ≤ -1] in the pairwise comparisons between tagatose-incubated samples and control samples at 4 DAI **(E)** and at 10 DAI **(F)** for each *Phytophthor*a spp.

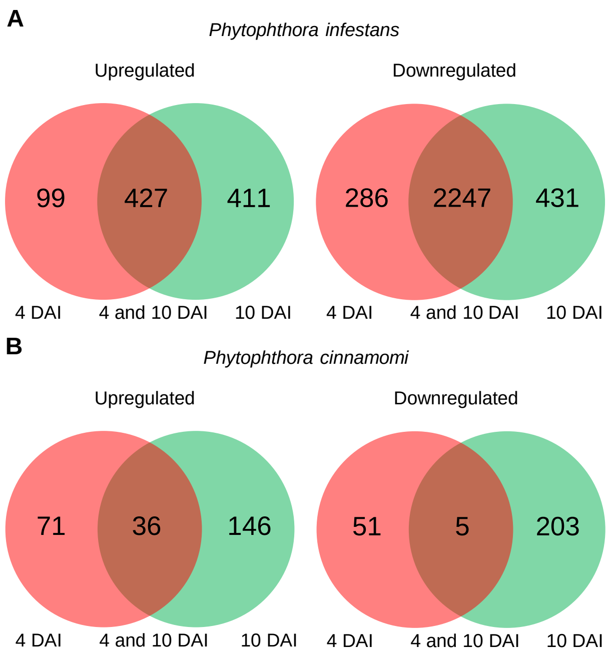

FIGURE S13 | Venn diagrams of genes modulated by tagatose in *Phytophthora* spp. Venn diagrams indicate the distribution of differentially expressed genes (DEGs) identified in *Phytophthora infestans* **(A)** and *P. cinnamomi* **(B)** at four and ten days after incubation (DAI) on pea agar medium in the presence of tagatose compared to the respective control samples grown in the absence of tagatose. DEGs of each *Phytophthora* spp. were grouped in genes significantly upregulated or downregulated at both time points (4 and10 DAI) and exclusively modulated at 4 DAI or at 10 DAI.

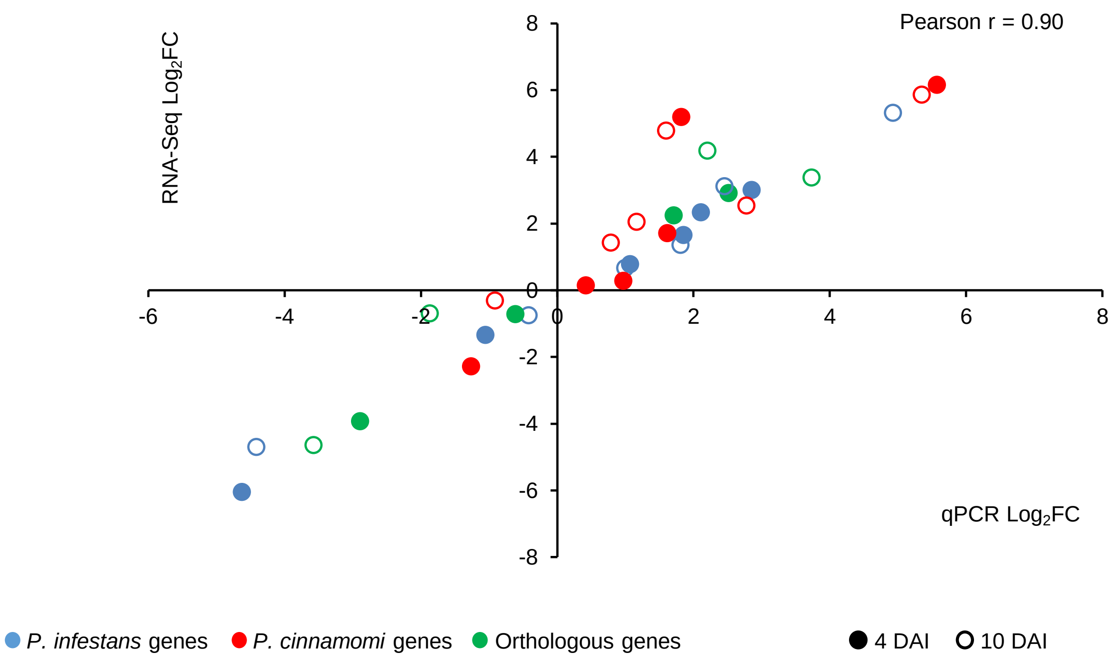

FIGURE S14 | Correlation analysis of gene expression data assessed by RNA-Seq and quantitative real-time PCR. Scatter plot and Pearson correlation value (r) on relative expression levels (Log_2-transformed fold change values) assessed using the RNA-Seq analysis and quantitative real-time PCR (qPCR) analysis of the selected *Phytophthora infestans* (blue) and *P. cinnamomi* (red) and *Phytophthora* orthologous (green) genes (Table S2) at four (solid circle) and ten (open circle) days after incubation (DAI) on pea agar medium in the presence of tagatose calculated as compared to the respective control samples grown in the absence of tagatose.
